# Factors associated with the delay in diagnosis of extrapulmonary tuberculosis at the patient and health system level: A study from a rural setting in India

**DOI:** 10.1371/journal.pone.0316273

**Published:** 2025-01-07

**Authors:** Shoaib Hassan, Reza Yaesoubi, Ole Bjørgaas Helle Magnus, Mala Kanthali, Manju Raj Purohit, Tehmina Mustafa

**Affiliations:** 1 Centre for International Health, Department of Global Public Health and Primary Care, University of Bergen, Bergen, Norway; 2 Yale School of Public Health, Yale University, New Haven, Connecticut, United States of America; 3 Department of Thoracic Medicine, Haukeland University Hospital, Bergen, Norway; 4 Department of Pathology, R.D. Gardi Medical College, Ujjain, India; 5 Department of Public Health Sciences, Karolinska Institute, Stockholm, Sweden; Hangzhou Red Cross Hospital, CHINA

## Abstract

**Background:**

With the proportion of tuberculosis cases that are extrapulmonary tuberculosis (EPTB) increasing in recent years, understanding and addressing factors contributing to the prolonged time to diagnosis (TTD) of EPTB patients is vital.

**Methods:**

We enrolled presumptive EPTB patients for a cohort study from 2018–2020 in Ujjain, India. Based on a structured questionnaire, the patients were interviewed for socio-demographic and clinical information, including previously visited health facilities (HF) for this illness. We analysed patients’ TTD, healthcare access, and referral pathways.

**Findings:**

EPTB (54%) and non-TB (58%) patients visited dispensaries during their first visit to a formal HF. Patients visited multiple HFs, including dispensaries (54%) and regional hospitals (32%), during 1–4 visits but did not receive an appropriate diagnosis. Less than 2% of the patients accessed private HFs. Most of the adult EPTB (83%) and non-TB (76%) patients were self-referred to our study site, where they were diagnosed. Our statistical models highlighted low-middle income groups, longer distances and longer travel time to HFs, and potentially less-empowered occupations as housewives with a prolonged TTD. Patients with a longer wait, including travel time, had a shorter TTD.

**Conclusion:**

We found individual, societal-level, and structural barriers to healthcare access and utilisation and their association with diagnostic delay among adult and paediatric EPTB patients.

## 1. Introduction

Among all tuberculosis (TB) case notifications, a high proportion (8–24%) of extrapulmonary tuberculosis (EPTB) patients are reported across all regions of the World Health Organization (WHO), including both medium-high and low-income countries [1, 2]. The challenges resulting from EPTB illnesses are beyond the patient level and can be felt at household- and societal levels [3]. Such illness-related consequences may worsen when the appropriate diagnosis is delayed. EPTB presents a multifaceted clinical landscape, displaying diverse signs and symptoms beyond the lungs. The disease manifestation depends on the affected body site, such as abdominal organs, lymph nodes, pleural tissues, parts of the central nervous system, and bones. Eventually, the illness exhibits a spectrum of disease manifestations and complexities that can simulate a range of illnesses [4, 5]. Many factors are associated with the late detection of EPTB patients [1, 6, 7]. Due to its non-contagious nature compared to pulmonary tuberculosis (PTB), the impact of EPTB is often overlooked within research and public health initiatives. As a result, the underestimation of EPTB’s significance and implications among the affected population results in delayed diagnosis, potentially leading to increased morbidity and challenges in effective disease management [6, 7]. Therefore, understanding and addressing factors contributing to delay in diagnosis of EPTB patients is vital for tackling disease management and reducing the overall disease burden.

Unlike PTB, globally, there is limited information about patient-related factors and their association with the delay in diagnosis for different manifestations of EPTB illness [6, 7]. For example, individual patient characteristics such as age may create unique barriers, as children and the elderly may face different challenges in accessing healthcare due to dependency issues or limited mobility [8–10]. Similarly, gender dynamics can affect healthcare access pathways, with societal expectations and cultural norms influencing the willingness of men and women to seek medical care for various health issues [11, 12]. Educational levels also play a crucial role in health-access behaviours, as individuals with lower education may have limited health literacy, impacting their understanding of symptoms and the importance of timely healthcare access [13]. The epidemiology of TB is rooted in disease awareness, social beliefs about this illness and cultural practices, including stigmas at a societal level. Such myriad factors may impact healthcare access, affecting a timely diagnosis [14].

The low awareness of EPTB symptoms among patients can lead to delays in healthcare access [14]. The available literature also indicates how structural barriers to accessing healthcare for EPTB patients, such as geographical disparities, lead to delayed or inadequate healthcare access [15, 16]. Additional factors such as employment status and occupational factors can also influence healthcare access [17]. Socioeconomic characteristics create significant barriers in healthcare-access pathways, with individuals from lower socioeconomic strata often facing financial constraints that limit their access to medical services [18]. Moreover, late diagnosis due to relatively less equipped health facilities regarding diagnostic tools or personnel can prolong an illness [6]. For example, collecting an EPTB specimen for diagnostic confirmation may require biopsy and fluid aspiration compared to a sputum sample of the PTB patient [19, 20]. All these factors may disproportionately affect relatively less focused EPTB patients, further widening the gap in timely diagnosis. A comprehensive evaluation of these distinct characteristics is essential for prompt intervention in EPTB. This pioneering study aimed to analyse factors impacting healthcare utilisation pathways and time to diagnosis (TTD) among children and adults with the objectives of (i) analysing factors impacting healthcare utilisation pathways and (iii) time to diagnosis (TTD) among children and adults with different manifestations of EPTB in a rural setting in India.

## 2. Methods

We conducted an institution-based prospective cohort study over two years, from November 2018 to February 2020, in Ujjain, Madhya Pradesh state of India. The study was based at the Chandrikaben Rashimikant Gardi Hospital (CRGH), associated with Ruxmaniben Deepchand Gardi Medical College (RDGMC) in Ujjain, Madhya Pradesh, India. This 750-bed referral and teaching hospital receives patients from semi-urban and rural areas. Patients could access this hospital directly or as referrals from public or private health facilities.

### 2.1. Data collection

We enrolled patients suspected of EPTB throughout the study period from the in and out-patient departments. Patients who had received anti-TB therapy during the last 12 months were excluded from the study. Each patient received a unique identifier to avoid the risk of duplication during the data collection. Following the pre-designed questionnaire, two trained hospital staff interviewed the enrolled patients. A specialist doctor participating in the study also randomly joined interviews to ensure adherence to set standards. Patients could respond in the national, Hindi, or local languages during the interview. Adult patients could respond themselves, while a caregiver provided information for children. We ensured patients’ privacy during the interview process.

The questionnaire (S2 Text) was reviewed by subject matter experts and contained questions for collecting demographic, socioeconomic, medical history, and information about the current illness. We transferred all the information from hard copies to the electronic version produced in Microsoft Excel. Two researchers randomly cross-checked 10% of the data entries for internal validation. After enrolment, we offered investigations and treatment to presumptive EPTB patients per the Indian National Tuberculosis Control Programme guidelines [21]. Qualified medical doctors conducted the relevant clinical examination and recorded their findings. The hospital pathology department collected biological specimens for laboratory confirmation. Additionally, EPTB patients were also clinically diagnosed based on the validated composite reference standard criterion [22]. After treatment initiation, EPTB patients were followed up for six months to monitor disease progression and effectiveness. Patients not starting ATT were followed until recovery or receipt of a diagnosis other than TB.

### 2.2. Operational definitions

**Fig 1:**
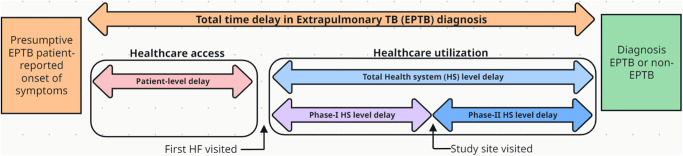
Illustrates the time to diagnosis (TTD) of extrapulmonary tuberculosis (EPTB) patients and respective delays in healthcare access and utilisation.

We defined time to diagnosis (TTD) as the interval from the onset of symptoms until an EPTB diagnosis (Fig 1). Patient-level TTD was the time from the onset of symptoms of the current illness until a patient first visited a health facility (HF). Health system-level TTD was the time interval between a patient’s first visit to HF and the diagnosis of current illness. Considering that all patients were diagnosed at our study site, we categorised health system (HS) level TTD in two phases. The duration of phase I of HS delay corresponded to the time between the patient’s first visit to a formal HF for EPTB signs & symptoms until they visited our study site. Phase II of HS delay was the duration between patients who first visited our study site until they were diagnosed with EPTB illness (positive or negative). The total HS-level TTD is constituted of HS-level phases I and II. The median of TTD was used as a cut-off to define a delay in diagnosis. A formal HF refers to any modern healthcare facility per the national health system, such as a dispensary, health centres, clinics, and public or private hospitals. To categorise patients for their EPTB and non-TB status, we used the composite reference standard (CRS), whose use for presumptive EPTB patients is established and also used in our study settings in Zanzibar [23–25]. Per the CRS, patients who were not classified as EPTB-positive were non-TB patients.

### 2.3. Socio-demographic and clinical disease characteristics

We categorised study participants into two groups: children and adults (older than 15 years). From patients’ interviews, we shortlisted potential factors that may impact TTD of presumptive EPTB illness. In line with the available literature and for additional analysis [12], we categorised study questions relevant to individual-, societal-level and structural barriers to understanding healthcare access and utilisation in the context of TTD. These factors include patients’ socio-demographic characteristics (age groups, gender, marital status, education level, occupation, and the number of family members), patients’ affordability, previous history of TB, any family history of TB if patients have heard of EPTB, self-medication for TB, any stigma about TB in patients’ society and disease manifestation. We used patients’ monthly salary categories (low, middle, and high) as a proxy indicator of their affordability to access healthcare.

### 2.4. EPTB patients’ healthcare utilisation

We studied healthcare accessing pathways of presumptive EPTB patients by subgroups, gender, patients’ affordability status, and disease manifestation. For this purpose, we analysed the types of HFs visited and the number of visits to the HFs until patients received an EPTB diagnosis (positive or negative) at our study site. Then, we produced a graphical mapping by the subgroups, as mentioned above. We analysed patients’ referral sources to the health facility to understand the referral pathways, eventually providing a diagnosis (our study site).

### 2.5. Data processing and statistical analysis

Firstly, we performed a descriptive analysis using the Chi-square test to identify statistically significant (p-value <0.05) differences among categorical variables. Secondly, we used univariable logistic regression to study potential factors associated with prolonged TTD. If we found an association (Odds Ratio (OR), p<0.25) during the univariable regression analysis, we considered those variables in the multivariable regression model. We presented results as adjusted odds ratios (aOR) with 95% confidence intervals (CI) and a p-value <0.05 as the statistically significant findings. We conducted all statistical analyses using R software employing the ggplot and tidyverse packages [26, 27].

### 2.6. Ethical approval

We obtained informed consent from all adult patients and parents or guardians of minors. As part of the clinical workup, before testing for HIV, both verbal and written consent were obtained. The study was approved by the Regional Committee for Medical Research Ethics in Norway (Ref: 2014/46/REK vest) and the Institutional Ethical Committee Ruxmaniben Deepchand Gardi Medical College, Ujjain, India (Ref: IEC 10/2018). Patients or the public were not involved in our research’s design, conduct, reporting, or dissemination plans.

## 3. Results

### 3.1. Socio-demographic and clinical characteristics

The presumptive EPTB patients enrolled in a prospective cohort study were classified as EPTB and non-TB patients based on the CRS, whose details are published elsewhere [24]. Our study results are based on the data from the interviews with 412 presumptive EPTB patients. Of these, 249 (60%) were classified as EPTB patients based on the CRS. Adult EPTB and non-TB patients had a median age of 28 years (Interquartile Range (IQR = 22–40)) and 38 years (IQR = 26–55), respectively. There were 55% female adult EPTB patients compared to 45% among non-TB patients (Table 1). The proportion of young-adult patients< 44 years old) was higher among EPTB patients (80%) compared to non-TB patients (58%). Overall, there were nine HIV-positive cases among our study cohort (of whom seven were among EPTB patients). Our descriptive analysis of EPTB patients is available in Table 1 & S1 Table 1 in S1 Text. Lymphadenitis was also the most frequent clinical manifestation among non-TB patients (65%), followed by meningitis (19%) and pleuritis (11%).

**Table 1:** Descriptive analysis of sociodemographic characteristics and individual-, societal-level and structural barriers to access to health care among presumptive EPTB patients.

			Adults			Children		
	Subgroups	Categories	EPTB patients	Non-EPTB patients	p-value*	EPTB patient	EPTB negative	p-value*
Individual (patient-level)	Age Groups (years)	15–24	85 (37.6)	27 (21.3)	<0.001	-	-	-
		25–44	95 (42.0)	47 (37.0)		-	-	-
		45–64	32 (14.2)	37 (29.1)		-	-	-
		>= 65	14 (6.2)	16 (12.6)		-	-	-
	Gender	Female	125 (55.3)	58 (45.7)	0.103	15 (65.2)	14 (38.9)	0.088
		Male	101 (44.7)	69 (54.3)		8 (34.8)	22 (61.1)	
	Marital status	Married	155 (68.9)	95 (74.8)	0.293	-	-	-
		Unmarried	70 (31.1)	32 (25.2)		-	-	-
	Affected body site (disease manifestations)	Lymphadenitis	132 (58.4)	79 (62.2)	0.010	20 (87.0)	27 (75.0)	0.421
		Pleuritis	49 (21.7)	17 (13.4)		1 (4.3)	1 (2.8)	
		Meningitis	20 (8.8)	24 (18.9)		1 (4.3)	7 (19.4)	
		Ascites	8 (3.5)	1 (0.8)		0 (0.0)	0 (0.0)	
		Others	17 (7.5)	6 (4.7)		1 (4.3)	1 (2.8)	
	Education levels	Primary or below	134 (59.3)	77 (60.6)	0.892	18 (78.3)	34 (94.4)	0.144
		Middle or Secondary	67 (29.6)	38 (29.9)		5 (21.7)	2 (5.6)	
		Higher	25 (11.1)	12 (9.4)		-	-	
	Occupation	Govt Employed	44 (19.5)	25 (19.7)	0.977	6 (26.1)	14 (38.9)	0.450
		Housewife	74 (32.7)	40 (31.5)		1 (4.3)	0 (0.0)	
		Unemployed	49 (21.7)	30 (23.6)		6 (26.1)	10 (27.8)	
		Private Employed	59 (26.1)	32 (25.2)		10 (43.5)	12 (33.3)	
	Salary Categories	Low income	27 (11.9)	20 (15.7)	0.596	3 (13.0)	3 (8.3)	0.831
		Middle income	61 (27.0)	32 (25.2)		8 (34.8)	14 (38.9)	
		High income	138 (61.1)	75 (59.1)		12 (52.2)	19 (52.8)	
	Number of family members	1–4	79 (35.0)	39 (30.7)	0.744	5 (21.7)	9 (25.0)	0.623
		5–7	106 (46.9)	66 (52.0)		14 (60.9)	21 (58.3)	
		8–10	33 (14.6)	19 (15.0)		4 (17.4)	4 (11.1)	
		11–20	8 (3.5)	3 (2.4)		0 (0.0)	2 (5.6)	
	Had TB previously	No	202 (89.4)	116 (91.3)	0.685	20 (87.0)	36 (100.0)	0.106
		Yes	24 (10.6)	11 (8.7)		3 (13.0)	0 (0.0)	
	Family History of TB	No	188 (83.2)	117 (92.1)	0.029	19 (82.6)	33 (91.7)	0.524
		Yes	38 (16.8)	10 (7.9)		4 (17.4)	3 (8.3)	
	Heard of TB	No	55 (24.3)	43 (33.9)	0.073	7 (30.4)	15 (41.7)	0.552
		Yes	171 (75.7)	84 (66.1)		16 (69.6)	21 (58.3)	
	Self-medication for TB	No	182 (80.5)	104 (81.9)	0.864	17 (73.9)	31 (86.1)	0.406
		Yes	44 (19.5)	23 (18.1)		6 (26.1)	5 (13.9)	
Community (societal level)	Stigma associated with TB	Yes/Uncertain	116 (51.3)	83 (65.4)	0.015	14 (60.9)	19 (52.8)	0.733
		No	110 (48.7)	44 (34.6)		9 (39.1)	17 (47.2)	
Structural barriers	First HF visited for this illness	Dispensary	122 (54.0)	71 (55.9)	0.719	12 (52.2)	24 (66.7)	0.275
		District Hospital	23 (10.2)	8 (6.3)		1 (4.3)	2 (5.6)	
		Health Center/Others	6 (2.7)	2 (1.6)		0 (0.0)	0 (0.0)	
		Private Hospital	3 (1.3)	2 (1.6)		2 (8.7)	0 (0.0)	
		Regional Hospital	72 (31.9)	44 (34.6)		8 (34.8)	10 (27.8)	
	Number of HFs visited	One	80 (35.4)	36 (28.3)	0.142	7 (30.4)	9 (25.0)	0.880
		Two	81 (35.8)	44 (34.6)		9 (39.1)	17 (47.2)	
		Three	14 (6.2)	5 (3.9)		1 (4.3)	1 (2.8)	
		Four	4 (1.8)	1 (0.8)		0 (0.0)	1 (2.8)	
		Missing	47 (20.8)	41 (32.3)		6 (26.1)	8 (22.2)	
	Number of visits to HFs	One	63 (27.9)	30 (23.6)	0.049	7 (30.4)	8 (22.2)	0.649
		Two	88 (38.9)	42 (33.1)		10 (43.5)	14 (38.9)	
		Three	22 (9.7)	19 (15.0)		2 (8.7)	4 (11.1)	
		Four	15 (6.6)	3 (2.4)		0 (0.0)	3 (8.3)	
		Missing	38 (16.8)	33 (26.0)		4 (17.4)	7 (19.4)	
	Travel time to the nearest HF	Below 30 min	81 (35.8)	58 (45.7)	0.049	8 (34.8)	18 (50.0)	0.465
		Between 30–60 min	97 (42.9)	54 (42.5)		8 (34.8)	11 (30.6)	
		Above 60 min	48 (21.2)	15 (11.8)		7 (30.4)	7 (19.4)	
	Travel time to this HF	Below 30 min	115 (50.9)	68 (53.5)	0.549	8 (34.8)	22 (61.1)	0.092
		Between 30–60 min	54 (23.9)	24 (18.9)		10 (43.5)	7 (19.4)	
		Above 60 min	57 (25.2)	35 (27.6)		5 (21.7)	7 (19.4)	
	Travel & Wait time to this HF	Below 10 min	103 (45.6)	65 (51.2)	0.254	16 (69.6)	24 (66.7)	0.471
		10–60 min	38 (16.8)	27 (21.3)		6 (26.1)	6 (16.7)	
		60–120 min	54 (23.9)	24 (18.9)		1 (4.3)	5 (13.9)	
		Above 120 min	31 (13.7)	11 (8.7)		0 (0.0)	1 (2.8)	

* p-value corresponds to the use of the Pearson chi-square test.

Among the EPTB patients, we came across several significant findings (p-value< 0.05). The most common disease manifestation was lymphadenitis (61%), pleuritis (20%), meningitis (8%), ascites (3%) and 7% undocumented (S1 Table 1 in S1 Text). Our data analysis across various EPTB manifestations revealed a significant difference (p-value< 0.05) based on several variables. There was a higher prevalence of young-adult EPTB patients (82%) in general and among lymphadenitis (90%), pleuritis (76%), and meningitis (67%) compared to those with ascites (50%). There was a higher proportion of females among EPTB patients (56%) and the ones having lymphadenitis (69%) or ascites (62%). Educational attainment of primary level or below was most common (>55%) across all subgroups. Among lymphadenitis and ascites patients, 55% and 75% were government-employed or housewives. A substantial majority (over 86%) had prior knowledge of TB. Stigma associated with TB was reported by 81% of meningitis patients and 75% of ascites patients. Furthermore, 34% of pleuritis and 24% of meningitis patients had more than two visits to healthcare facilities. A significant number of pleuritis (80%) and meningitis (86%) patients experienced prolonged travel and wait times to reach the HF providing diagnosis, our study site.

Our study cohort included 23 and 36 children among EPTB and non-TB patients, respectively. Within the children subset, the median age was nine years (IQR = 6–11 years), including 65% and 39% female patients among EPTB and non-TB patients, respectively (S1 Table 1 & 2 in S1 Text). The most common disease manifestations were lymphadenitis (87%), pleuritis (4.3%), and meningitis (4.3%). We identified a significantly higher (p-value < 0.05) stigma associated with TB within the community settings of lymphadenitis and meningitis patients (51% and 88%, respectively). Of the eight meningitis patients, five (63%) experienced travel and wait times exceeding 60 minutes to reach the HF providing diagnosis, our study site (S1 Table 1 in S1 Text).

### 3.2. Healthcare utilisation pathways among adults

Our study cohort has the most common first contact with the health system at dispensaries: (ETPB, 54% and non-TB, 58%). A regional hospital followed this in the area as the second most common health facility accessed by study participants (EPTB, 32% and non-TB, 22%). Less than 2% of the patients accessed private HFs. None of the patients with low income visited private HFs. A smaller proportion of patients contacted the district hospital (8.3%) and health centre (2%) (S1 Table 1 & 3 in S1 Text). For the type of first health facility visited, our study cohort had no significant difference (p-value >0.05) based on subgroups such as gender, affordability, and disease manifestation (S1 Table 3 in S1 Text).

**Fig 2:**
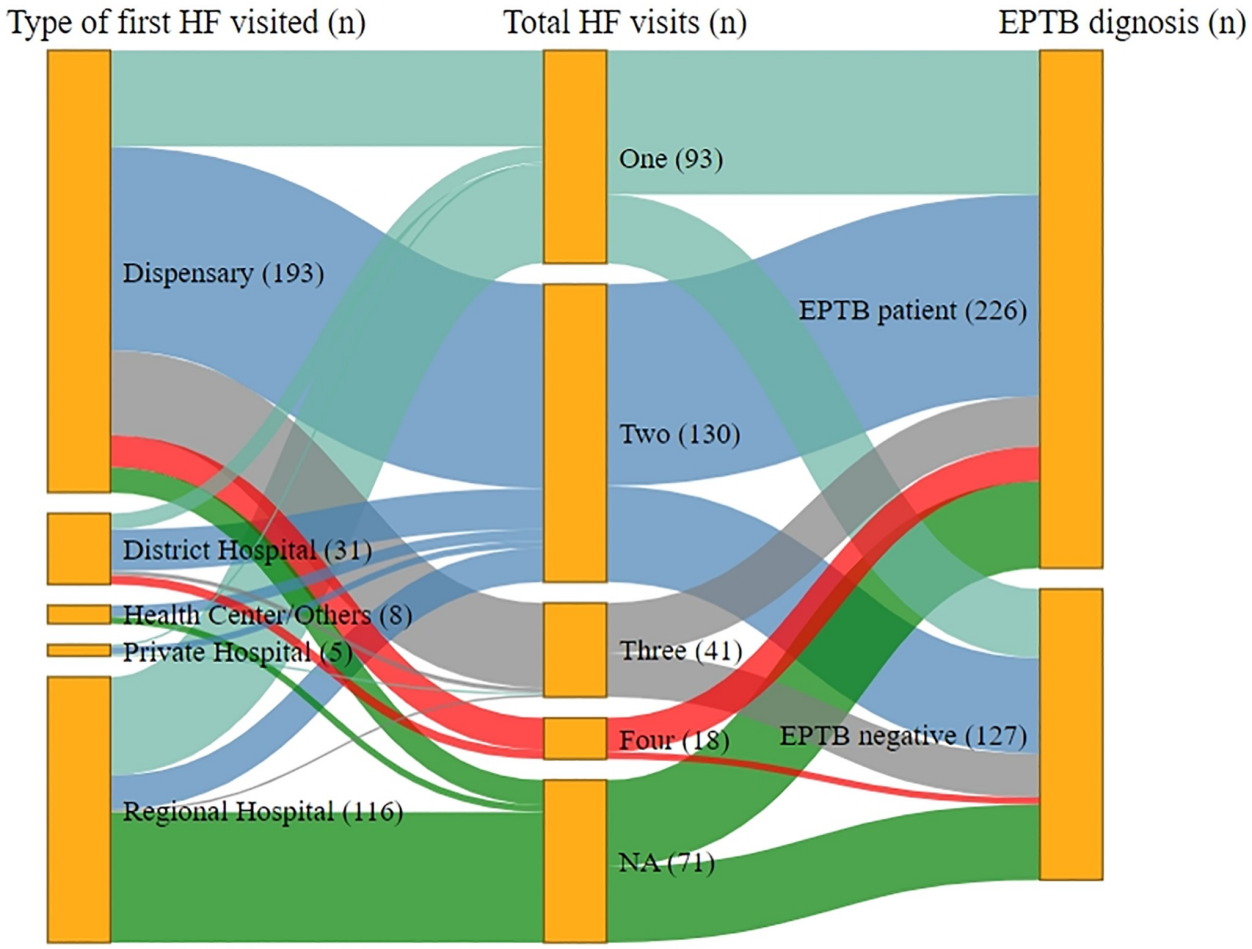
Illustrations of healthcare access pathways followed by presumptive EPTB patients among adults.

**Fig 3:**
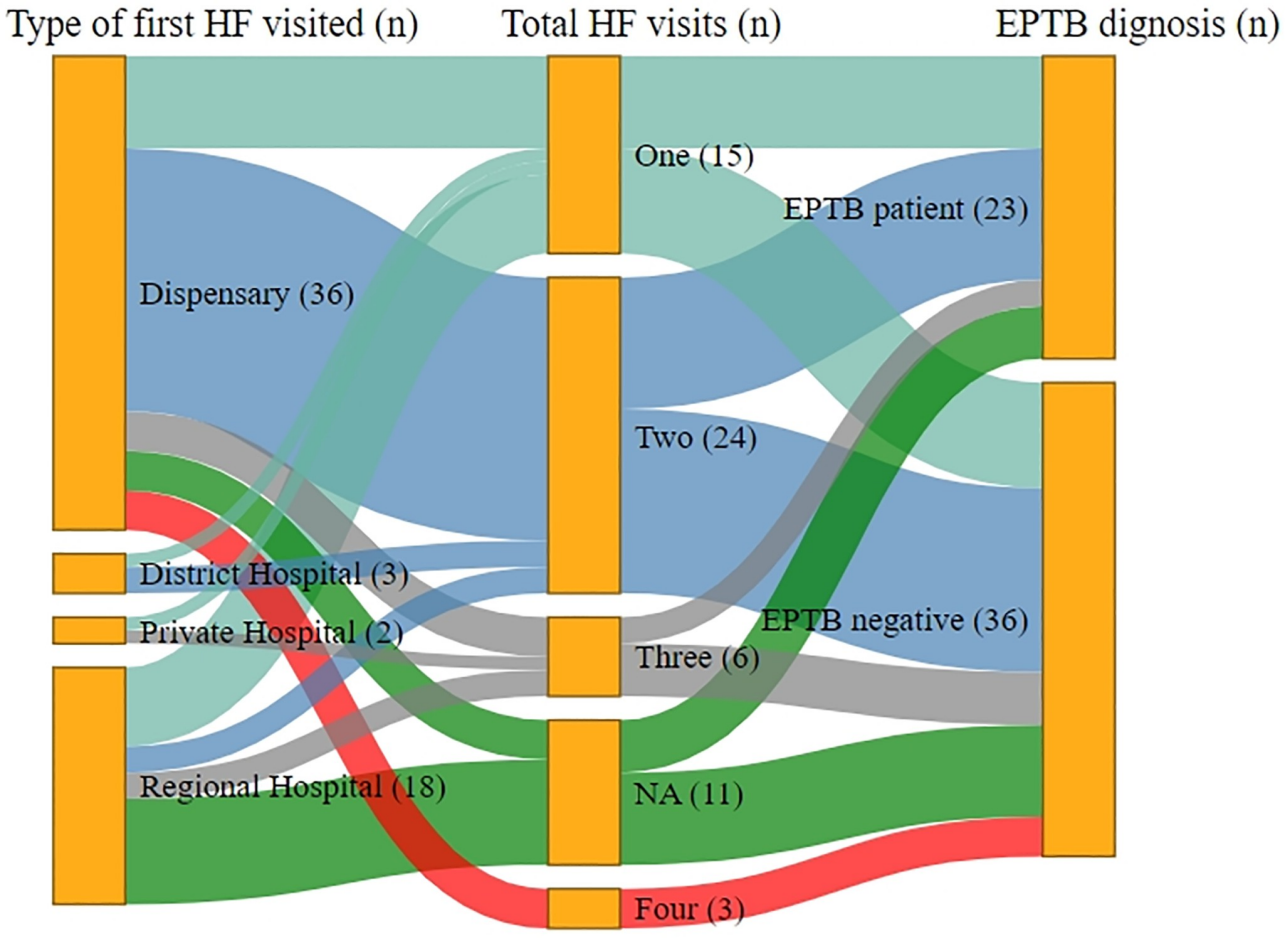
Illustrations of healthcare access pathways followed by presumptive EPTB patients among children.

In general, both EPTB and non-TB patients sought care from multiple health facilities (median 1, range 1–4) and reported frequent visits to HFs (median 2, range 1–4). However, these HF-visits did not translate into appropriate diagnoses until patients contacted our study site. Categorised by adults and children, we share a graphical representation and descriptive analysis of the types of health facilities accessed against the total number of visits to access healthcare as shown in Figs 2 and 3. We did not find significant differences between adults and children in this healthcare access (p-value >0.05).

Furthermore, Fig. A-E in S1 Text illustrates the graphical mapping and descriptive analysis of HFs accessed against the total number of healthcare visits categorised by gender (Fig. A and B in S1 Text), affordability status (Fig. C-E in S1 Text), and disease manifestation (Fig. F-H in S1 Text). There was a significant difference (p-value <0.05) among subgroups based on gender (female & male), affordability status (low, middle & high income), and EPTB manifestation (lymphadenitis, pleuritis & meningitis). For healthcare access, presumptive EPTB patients predominantly visited dispensaries, followed by a regional hospital for their first visit to an HF (S1 Table 4 in S1 Text). Until four HF visits, the dispensary was the most common HF visited by the analysed subgroups of presumptive EPTB patients. Among meningitis patients, visiting the district hospital was also common on their fourth HF-visit (66%).

We also conducted an analysis of total visits to HFs. Low- and middle-income groups had a higher proportion of patients with 3–4 total HF-visits compared to the high-income group with higher 1–3 HF-visits (p-value <0.05) (S1 Table 5 in S1 Text). The lymphadenitis patients had a lower (1–2) number of total HF visits compared to those with pleuritis with 3–4 HF visits (p-value <0.05). Most patients (EPTB, 83% and non-TB, 76%) contacted the study site on their own initiative without a referral from a primary-care level HF. A few EPTB patients were referred to the study site by private HF (5.7%), family members (5.7%), and any governmental HF (1.4%). Among the non-TB patients, common sources of referral were private HF (15.8), a family member (2.6%) or a governmental HF (2.6%) (S1 Table 6 in S1 Text).

### 3.3. Healthcare utilisation pathways among children

Like adults, dispensaries were the first HF visited among the children to access healthcare (S1 Table 1 in S1 Text). Most of the patients accessing our study site were either self-referred (73%) or referred by members of a family (6.7%) or a governmental HF (6.7) (p-value >0.05) (S1 Table 6 in S1 Text).

### 3.4. Time to diagnosis (TTD) and factors associated with diagnostic delay among adults

**Fig 4:**
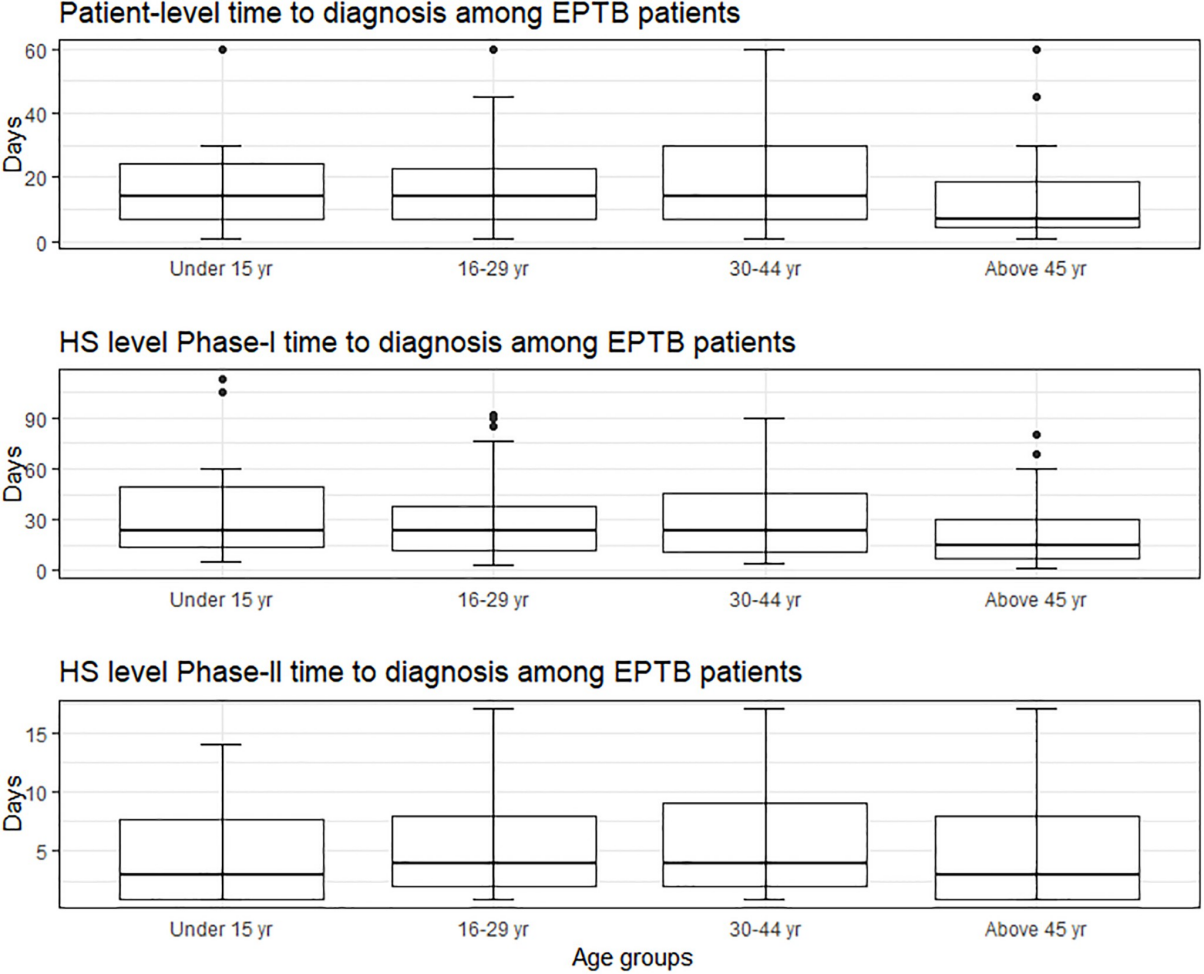
Time to diagnosis among the cohort of confirmed EPTB patients by age groups (excluding outliers and upper limit of the delay (number of days) at 90^th^ percentile).

**Fig 5:**
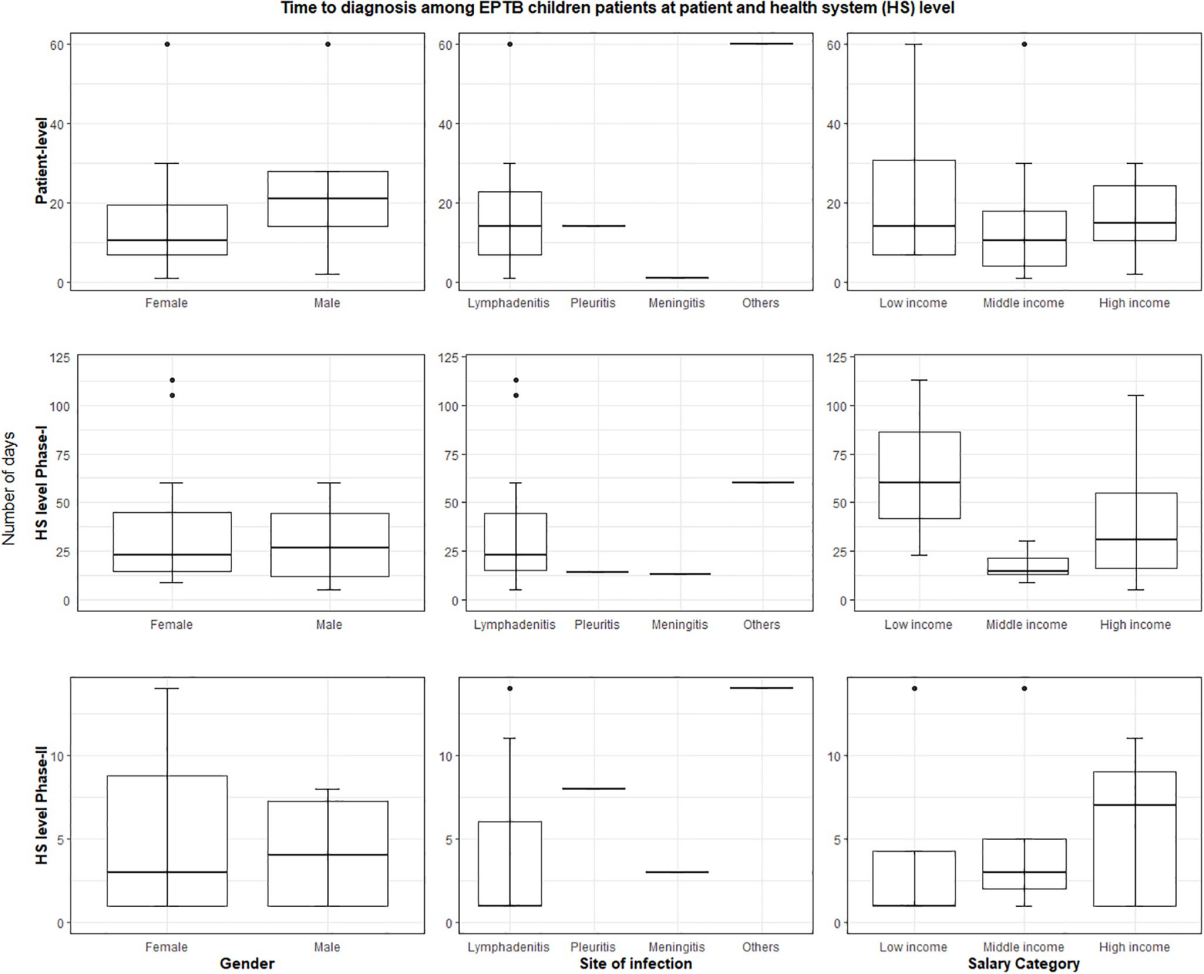
Time to diagnosis of confirmed EPTB patients among children (excluding outliers and upper limit of the delay (number of days) at 90^th^ percentile).

**Fig 6:**
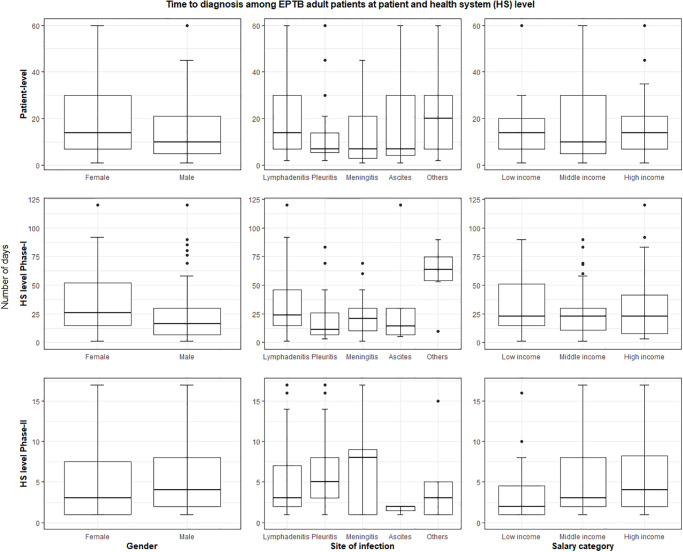
Time to diagnosis of confirmed EPTB patients among adults (excluding outliers and upper limit of the delay (number of days) at 90^th^ percentile).

Among our study cohort, the median TTD at the patient level was 14 days (IQR = 7–30, range = 7–1095). At HS level, the median TTD during phase-I was 15 days (IQR = 4–39 days, range = 1–3620 days) and four days (IQR = 1–9, range = 1–59) during phase-II. The median TTD comprising both at the patient- and HS- levels (phase I and II) was 21 days (IQR = 8–48, range 1–3622). The descriptive summary measures of patient and HS-level delays among various age groups and subgroups based on gender, EPTB disease manifestations and affordability status among children or adult EPTB patients are shown in Figs 4–6. We used median TTD as the cut-off threshold to label TTD as prolonged delays at the patient- and HS levels. Then, we performed a multivariable regression analysis to study the factors significantly associated with prolonged (above median) TTD (S1 Table 7 in S1 Text).

**Fig 7:**
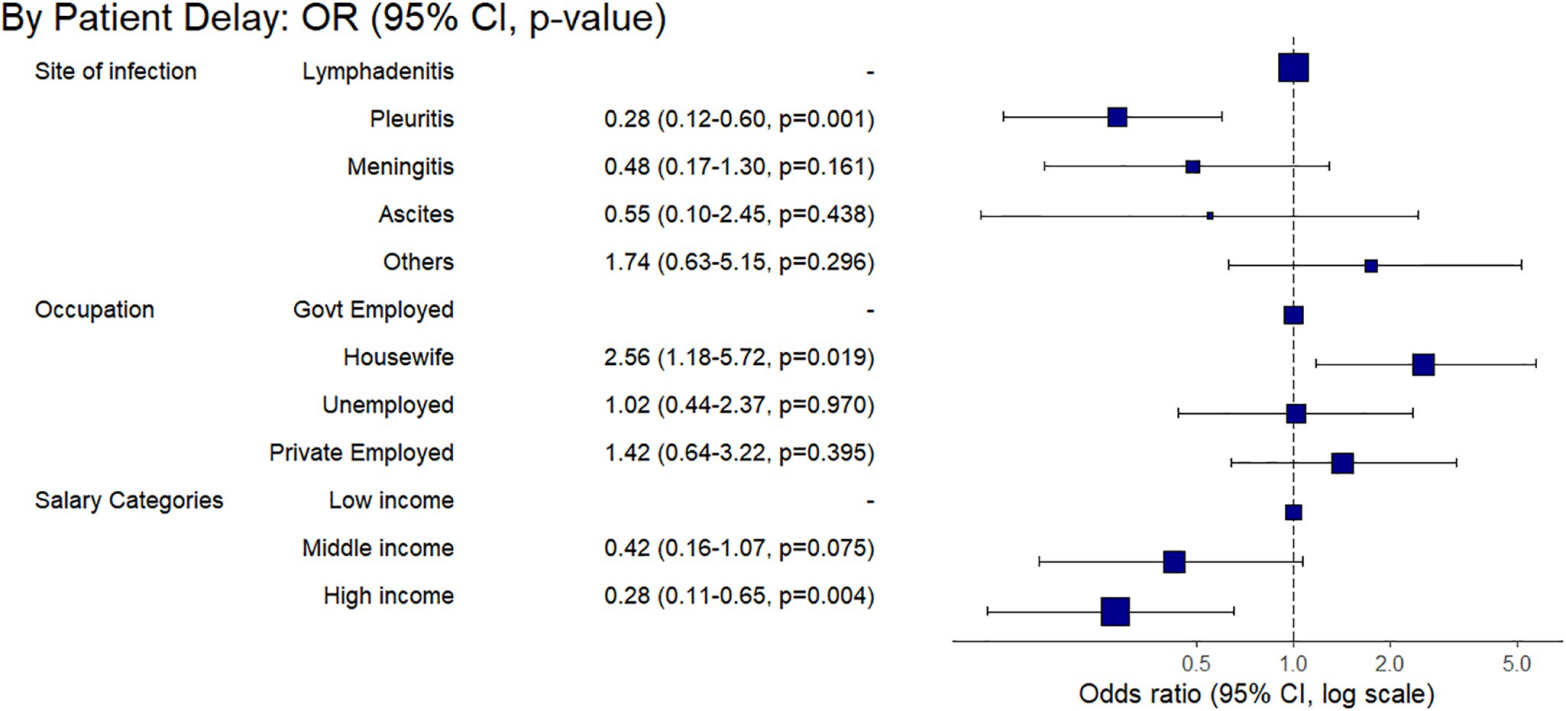
Factors associated with the prolonged patient-level delay based on multivariable final regression model among the adult group of confirmed EPTB patients.

**Fig 8:**
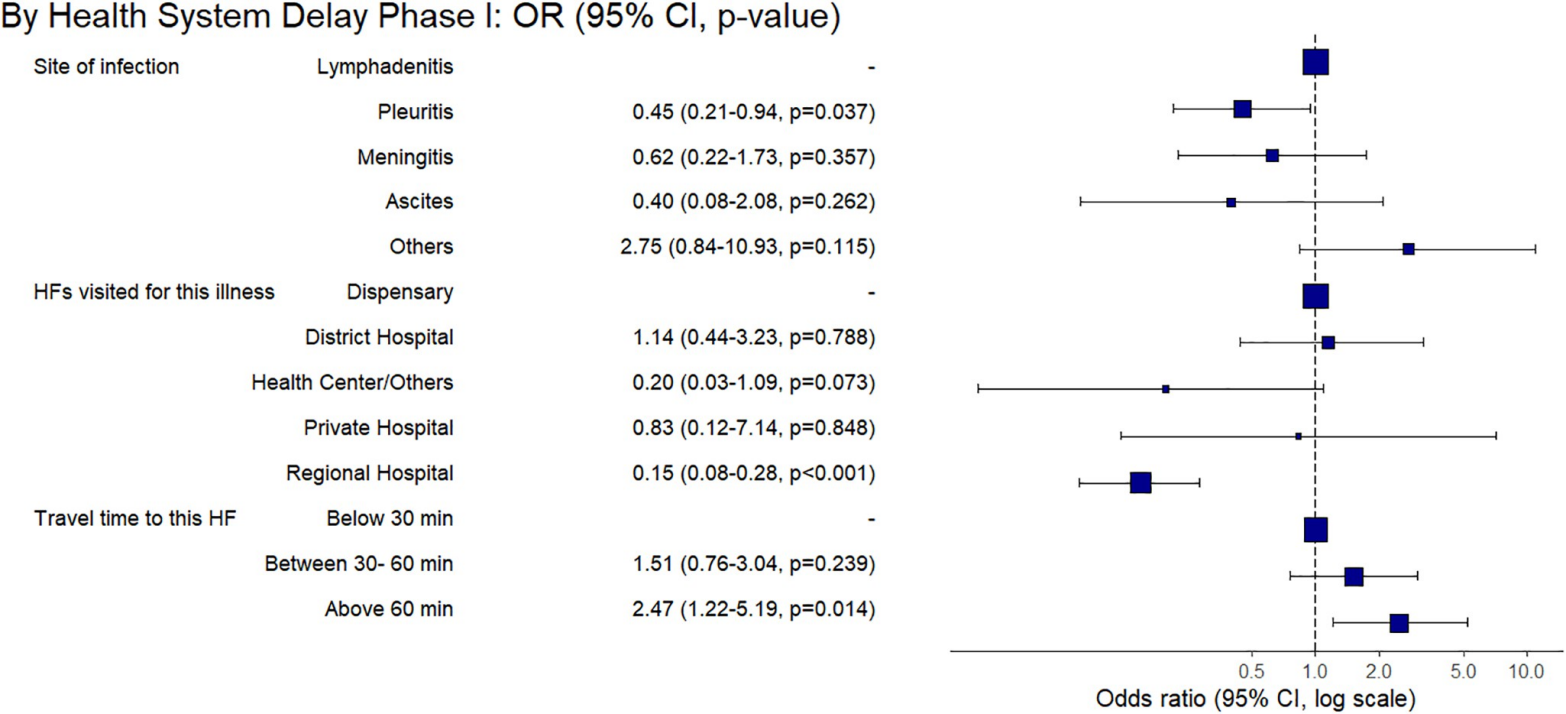
Factors associated with the prolonged health system-level phase-I delay based on multivariable final regression model among the adult group of confirmed EPTB patients.

**Fig 9:**
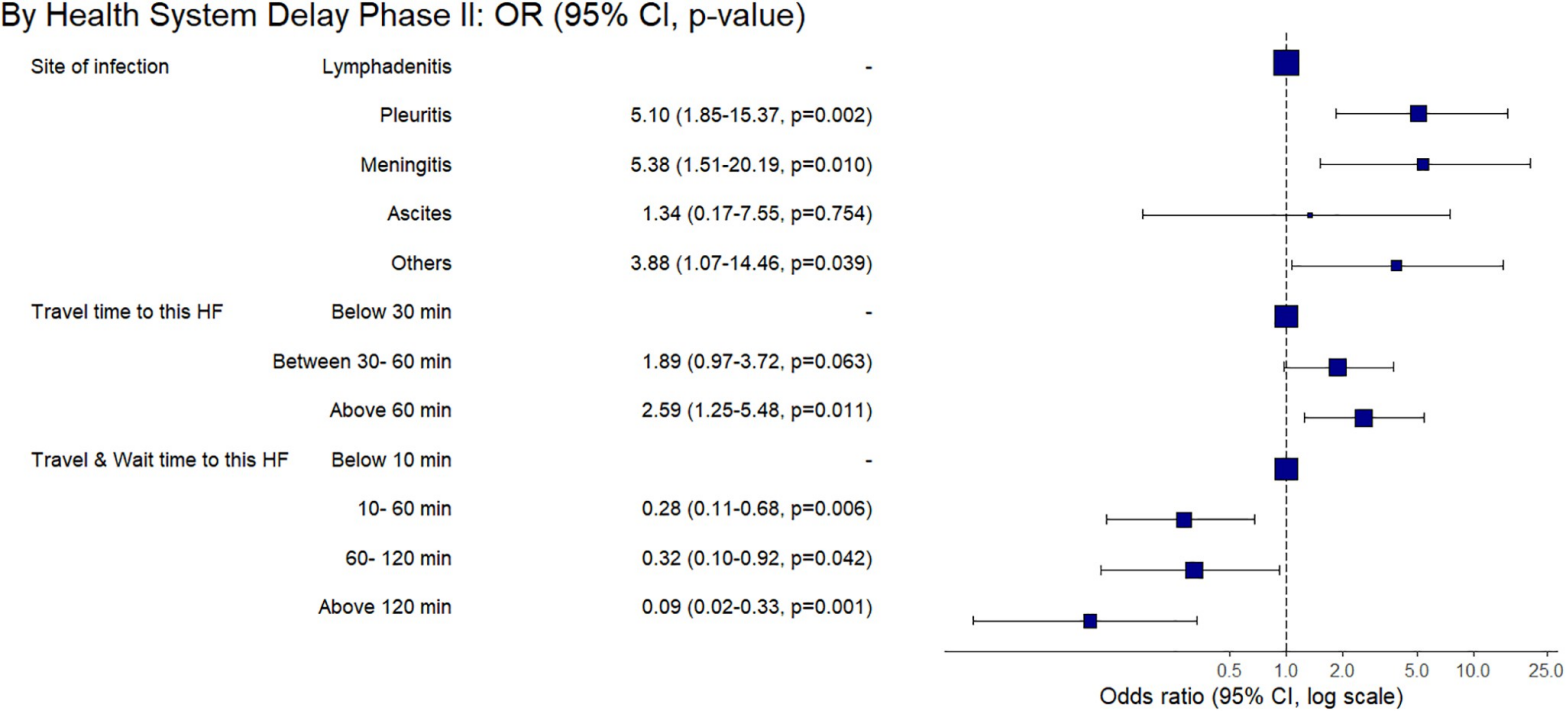
Factors associated with the prolonged health system-level phase-II delay based on multivariable final regression model among the adult group of confirmed EPTB patients.

Based on the multivariable regression analysis of confirmed EPTB patients’ data, we built final regression models highlighting factors significantly associated with the prolonged delays at the patient- and HS levels (Figs 7–9). Among the ETPB patients, we found significantly prolonged patient-level delay by the ones reporting housewife as an occupation (aOR 2.56, 95% CI 1.18–5.72) compared to the government employees. While compared to lymphadenitis patients, ones having pleuritis had a shorter patient-level delay (aOR 0.28, 95% CI 0.12–0.60). We also found shorter patient-level delays among high-income groups (aOR 0.28, 95% CI 0.11–0.65) compared to the low-income group (Fig 7).

For the phase-I HS-level, patients with pleuritis had a shorter delay (aOR 0.45, 95% CI 0.21–0.94) compared to the lymphadenitis patients. Similarly, patients accessing a regional hospital in the area as their first contact with health care had a shorter delay (aOR 0.15, 95% CI 0.08–0.28) than those accessing dispensaries. Longer (>60 minutes) travel time to our study site was associated with phase-I HS-level prolonged delay (aOR 2.47, 95% CI 1.22–5.19) as compared to the travel time below 10 minutes (Fig 8).

The phase-II HS-level, unlike phase 1, patients with pleuritis (aOR 5.10, 95% CI 1.85–15.37) and meningitis (aOR 5.38, 95% CI 1.51–20.19) faced a prolonged delay compared to the ones having lymphadenitis. A travel time of more than >60 minutes to our study site was also marginally associated with the prolonged delay (aOR 2.59, 95% CI 1.25–5.48) compared to the travel time below 10 minutes. However, compared to a combined travel & wait time of <10 minutes, the ones having more extended travel and wait time of 10–60 minutes, 60–120 minutes, and >120 minutes were less likely to be associated with the longer phase-II HS-level delay (aOR 0.28, 95% CI 0.11–0.68, aOR 0.32, 95% CI 0.10–0.92 and (aOR 0.09, 95% CI 0.02–0.33, respectively). Thus, the ability to travel towards HF, which provided diagnosis and a longer wait, reduced the phase-II health-system level delay (Fig 9).

### 3.5. Time to diagnosis (TTD) and factors associated with diagnostic delay among children

**Fig 10:**
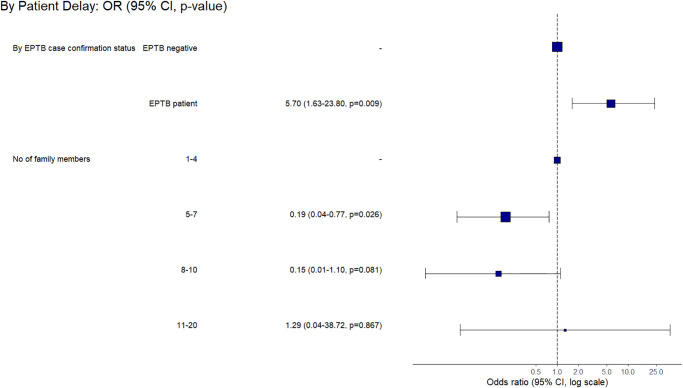
Factors associated with the prolonged patient-level delay based on multivariable final regression model among the children group of confirmed EPTB patients.

**Fig 11:**
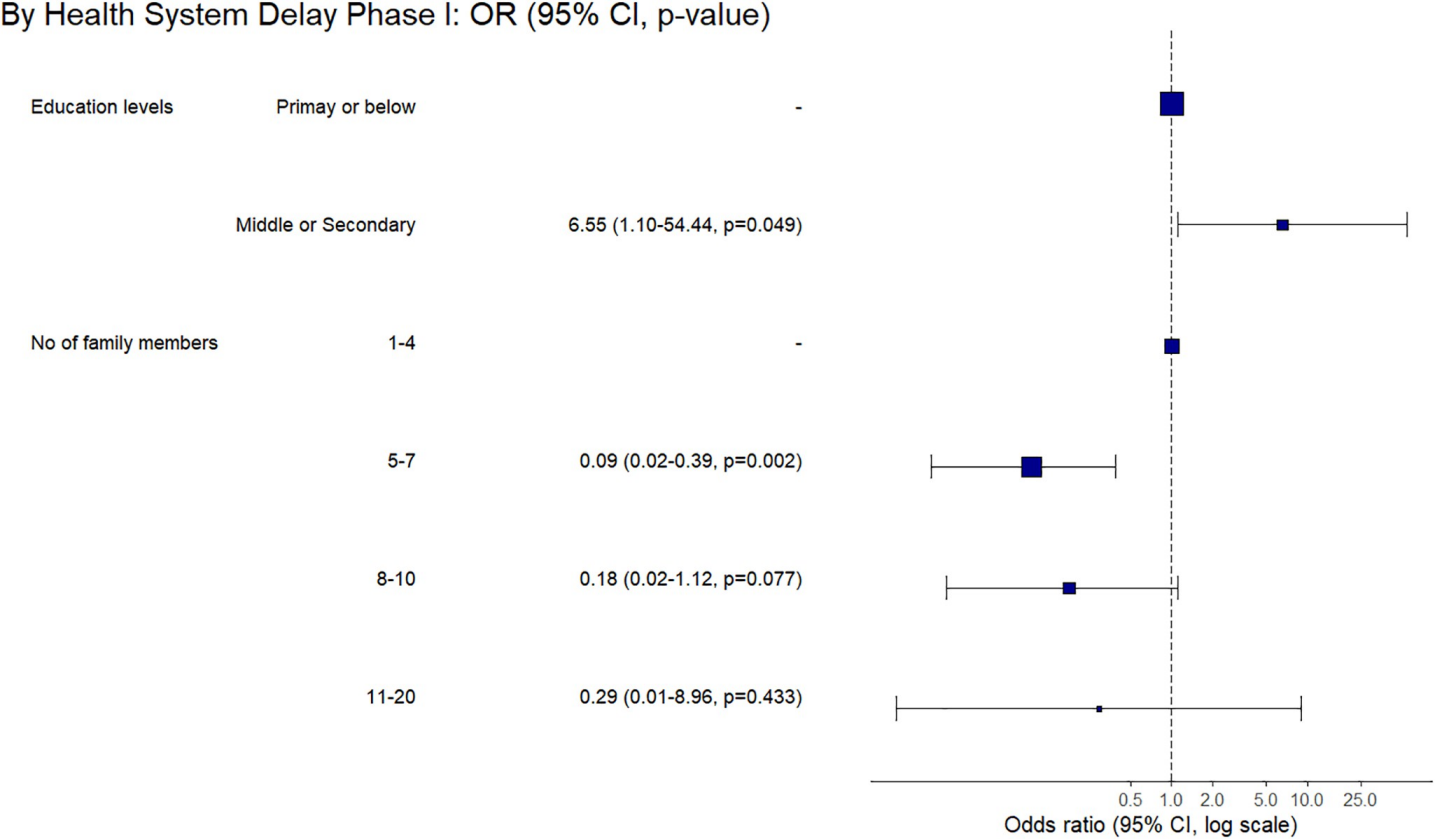
Factors associated with the prolonged health system-level phase-I delay based on multivariable final regression model among the children group of confirmed EPTB patients.

Among presumptive EPTB patients in sub-cohort of children too, we found factors associated with prolonged patient and HS-level delays based on multivariable regression analysis (S1 Table 8 in S1 Text elaborates on various types of delays among children). The final regression model showed that, unlike adults, the EPTB patients among children faced a prolonged patient-level delay (aOR 5.70, 95% CI 1.63–23.80) compared to the non-TB patients (Fig 10). While compared to the children from a family size of 1–4 members, the ones from 5–7 had a lower likelihood (aOR 0.19, 95% CI 0.04–0.77) of prolonged patient-level delay (Fig 10). The phase I HS-level prolonged delay was also less likely (aOR 0.09, 95% CI 0.02–0.39) among children from a family size of 5–7 compared to 1–4 members (Fig 11). At the same time, children who had completed middle or secondary school were associated with prolonged phase-I HS-level delay (aOR 6.55, 95% CI 1.10–54.44) compared to the ones having education levels of primary or below. We found no factors associated with a prolonged phase-II HS-level delay (S1 Table 8 in S1 Text).

## 4. Discussion

This study has analysed individual, societal-level, and structural barriers to healthcare access and utilisation as well as factors associated with diagnostic delays among adult and paediatric EPTB patients. Housewives faced a longer patient-level delay in accessing healthcare than government-employed patients. This finding implies a financial dependency on the economically active head of the household. Such cultural and gender dynamics are known to play a role in societal norms by influencing women’s healthcare-accessing patterns for EPTB, either due to stigma or dependence on male decision-makers within households [28]. We also found that high-income groups were less at risk of prolonged delay compared to low-income. Dispensaries offer basic medical services and preventive care in semi-urban and rural areas. These findings reflect how patients’ affordability becomes a potentially decisive factor in accessing healthcare, especially those suffering from chronic diseases such as EPTB. The association between poverty and delay in diagnosis is known for tuberculosis patients [18]. As per existing literature, we also found that income significantly influences access to healthcare, and lower income levels often correlate with limited access to healthcare facilities and diagnostic services [29]. This highlights income-related disparities in healthcare access for EPTB patients and emphasises the need for targeted interventions to mitigate financial obstacles and improve overall accessibility irrespective of economic status and affordability [18]. Children from medium-sized families (5–7 members) compared to the smaller (1–4 members) families were less likely to be associated with prolonged patient-level delay. This shows how minors depend on caregivers to access healthcare, and large families might have more caregivers. Thus, factors such as being from low-middle socioeconomic groups, housewives that are culturally less empowered for decision-making in our study setting, and dependence on caregivers such as children led to facing prolonged TTD.

The limited literature about the diagnostic delays of EPTB patients prevents a broader comparison with existing literature. However, a couple of studies reported a prolonged TTD among EPTB patients. Their reported median patient and health system-level delay was 42 days in Norway [30]. On the other hand, a patient and health system delay of 8 weeks and seven weeks was reported in India [7]. We found a relatively shorter than the ones reported in these studies. This may be due to improved patients’ knowledge and behaviour about the EPTB disease or an active follow-up of our study participants as part of our study settings. To our knowledge, no study has reported factors associated with various types of diagnostic delays among EPTB patients, as elaborated in our study.

We found several factors influencing health system-level delays in TTD. For example, sub-optimal healthcare accessibility due to long travel time to the HFs giving diagnosis services becomes a structural barrier, affecting healthcare utilisation thus, a prolonged TTD. However, we found that patients who could wait longer at well-equipped diagnostic facilities, like our study site, received diagnostic confirmation. Hence, being able to wait longer could be a decisive factor in the timely diagnosis of EPTB. Such findings are in line with the existing literature [5]. Thus, if EPTB patients cannot wait to complete the diagnostic process at a well-equipped HF, they may face a prolonged EPTB illness. The PTB-like routine and structured follow-up of EPTB patients after their first contact with the HFs may address such a challenge. Otherwise, several HF encounters initiated by patients did not lead to appropriate diagnosis nor referral to better-equipped HFs. However, we found that a regional hospital, also deemed relatively well-equipped, prevented a prolonged HS-level phase-I delay. Thus, if health systems are strengthened and accessible to EPTB patients, it can lead to a shorter TTD. Moreover, a shorter patient-level and HS-level phase-I delay among patients with pleuritis compared to lymphadenitis shows how a higher suspicion of PTB-like symptoms led patients to seek healthcare earlier and earlier diagnosed. Otherwise, patients with a relatively low prevalence of EPTB manifestation, such as meningitis, had a prolonged HS-level phase-II delay despite its severity. However, this may be due to non-specific EPTB manifestations leading to a varying subjective suspicion of EPTB illness among healthcare providers and challenging diagnostic procedures for EPTB site infections. Thus, despite patients’ encounters with the health system, the opportunity to diagnose EPTB is not ensured at the HS level.

Our study has some limitations. We did not have additional data about each previous visit a patient made to HFs, qualitative data, and the kind of diagnostic interventions that took place. This information could have helped us highlight any additional challenges faced by patients. As part of our cohort study, we actively followed the diagnostic results of presumptive EPTB patients, which may affect the generalizability of study results. In real-life scenarios, such an active follow-up of diagnostic results may not happen, leading to even a prolonged TTD. Due to the relatively smaller proportion of children, we could not run additional analyses for this subgroup. We did not follow up on non-TB patients for their diagnosis, nor did we have information about other non-tuberculosis mycobacterial illnesses among our study cohort. Our findings are based on a single study site from self-reported patients’ information, which may limit the generalisability of findings in some cases.

## 5. Conclusion

In conclusion, factors leading to prolonged TTD at the patient- and health system levels vary. Patients reported a delay in access to healthcare for less severe disease manifestations such as lymphadenitis and longer travel time to HFs. However, once patients accessed the health system, there were missed opportunities for diagnosing them unless they could wait longer at a well-equipped HF. In the absence of routine follow-up of such patients, even multiple encounters with HFs did not lead to timely diagnosis, hence, a prolonged EPTB illness. It is likely that during patients’ visits to relatively less-equipped HFs, they received symptomatic treatment until patients attended our study site. Therefore, following EPTB patients’ access to healthcare, a systematic follow-up policy of presumptive EPTB should be ensured. In future, EPTB disease awareness, diagnostic services and referral to well-equipped HFs should be ensured at frequently visited less-equipped HFs closer to communities. This can be ensured by strengthening primary healthcare services under universal health coverage.

## Supporting information

S1 Text S1 Fig. A. Illustrations of healthcare access pathways followed by presumptive EPTB patients among females. S1 Fig. B. Illustrations of healthcare access pathways followed by presumptive EPTB patients among males. S1 Fig. C. Illustrations of healthcare access pathways followed by presumptive EPTB patients among low-income groups. S1 Fig. D. Illustrations of healthcare access pathways followed by presumptive EPTB patients among middle-income group. S1 Fig. E. Illustrations of healthcare access pathways followed by presumptive EPTB patients among high-income groups. S1 Fig. F. Illustrations of healthcare access pathways followed by presumptive EPTB patients having lymphadenitis. S1 Fig. G. Illustrations of healthcare access pathways followed by presumptive EPTB patients having pleuritis. S1 Fig. H: Illustrations of healthcare access pathways followed by presumptive EPTB patients having meningitis. S1 Table 1: Descriptive analysis of sociodemographic characteristics and individual-, societal-level and structural barriers in access to health care among presumptive EPTB patients based on site of infection. S1 Table 2: Presumptive EPTB patients’ characteristics among children based on sites of infection as disease manifestation. S1 Table 3: Health facilities accessed by subgroups of presumptive EPTB patients for their first access to healthcare. S1 Table 4: Subgroup analysis of presumptive EPTB patients’ access to health facilities against the total number of visits. S1 Table 5: Subgroup analysis of confirmed EPTB patients’ total number of HF visits. S1 Table 6: A descriptive analysis of EPTB patients’ first HF visit against referral to the study site that provided EPTB diagnostic confirmation. S1 Table 7: Factors associated with prolonged time to diagnosis among confirmed EPTB adult patients. S1 Table 8: Factors associated with prolonged time to diagnosis among children presumptive EPTB patients.
(PDF)

S2 Text Study questionnaire. (PDF)
