# Supplementary material for: Factors associated with the delay in diagnosis of extrapulmonary tuberculosis at the patient and health system level: A study from a rural setting in India
Source: PLoS One. 2025 Jan 7;20(1):e0316273. doi: 10.1371/journal.pone.0316273 (PMC11706485; doi:10.1371/journal.pone.0316273)
Supplement: S1 Text — S1 Fig. A. Illustrations of healthcare access pathways followed by presumptive EPTB patients among females. S1 Fig. B. Illustrations of healthcare access pathways followed by presumptive EPTB patients among males. S1 Fig. C. Illustrations of healthcare access pathways followed by presumptive EPTB patients among low-income groups. S1 Fig. D. Illustrations of healthcare access pathways followed by presumptive EPTB patients among middle-income group. S1 Fig. E. Illustrations of healthcare access pathways followed by presumptive EPTB patients among high-income groups. S1 Fig. F. Illustrations of healthcare access pathways followed by presumptive EPTB patients having lymphadenitis. S1 Fig. G. Illustrations of healthcare access pathways followed by presumptive EPTB patients having pleuritis. S1 Fig. H: Illustrations of healthcare access pathways followed by presumptive EPTB patients having meningitis. S1 Table 1: Descriptive analysis of sociodemographic characteristics and individual-, societal-level and structural barriers in access to health care among presumptive EPTB patients based on site of infection. S1 Table 2: Presumptive EPTB patients’ characteristics among children based on sites of infection as disease manifestation. S1 Table 3: Health facilities accessed by subgroups of presumptive EPTB patients for their first access to healthcare. S1 Table 4: Subgroup analysis of presumptive EPTB patients’ access to health facilities against the total number of visits. S1 Table 5: Subgroup analysis of confirmed EPTB patients’ total number of HF visits. S1 Table 6: A descriptive analysis of EPTB patients’ first HF visit against referral to the study site that provided EPTB diagnostic confirmation. S1 Table 7: Factors associated with prolonged time to diagnosis among confirmed EPTB adult patients. S1 Table 8: Factors associated with prolonged time to diagnosis among children presumptive EPTB patients. (PDF) [file pone.0316273.s001.pdf]

**Fig. A. Illustrations of healthcare access pathways followed by presumptive EPTB patients among females.**

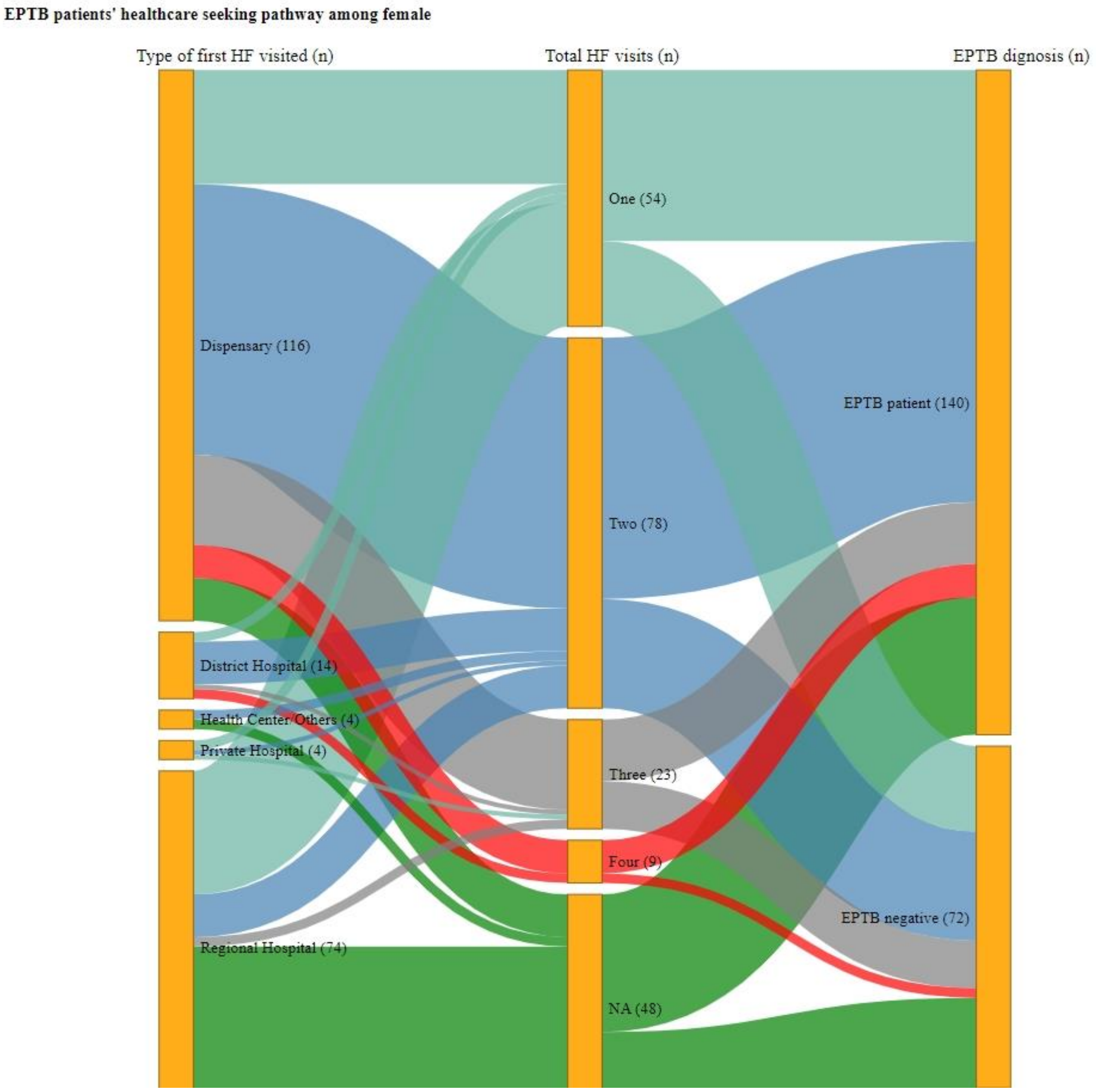

**Fig. B. Illustrations of healthcare access pathways followed by presumptive EPTB patients among males.**

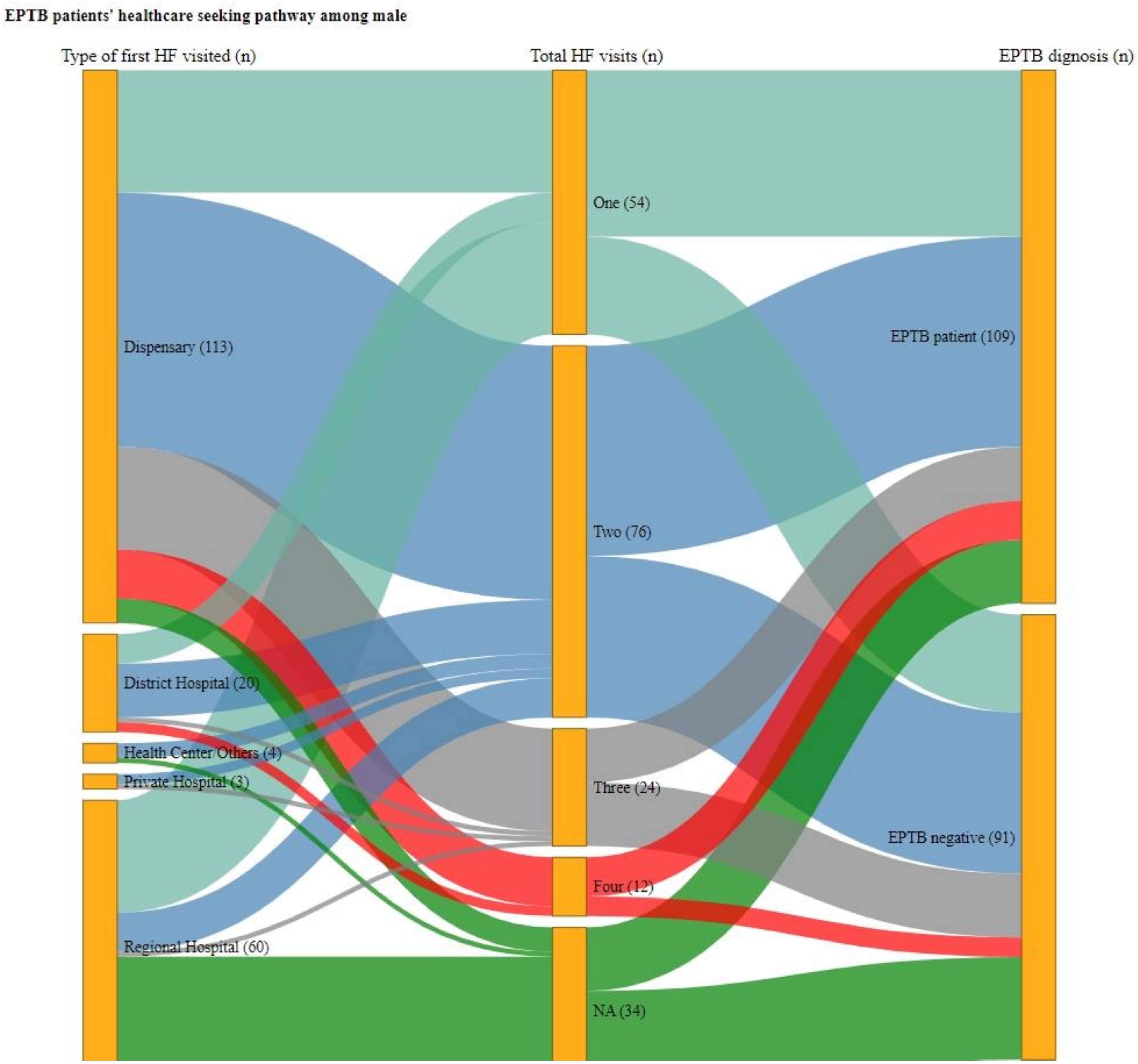

**Fig. C. Illustrations of healthcare access pathways followed by presumptive EPTB patients among low-income groups.**

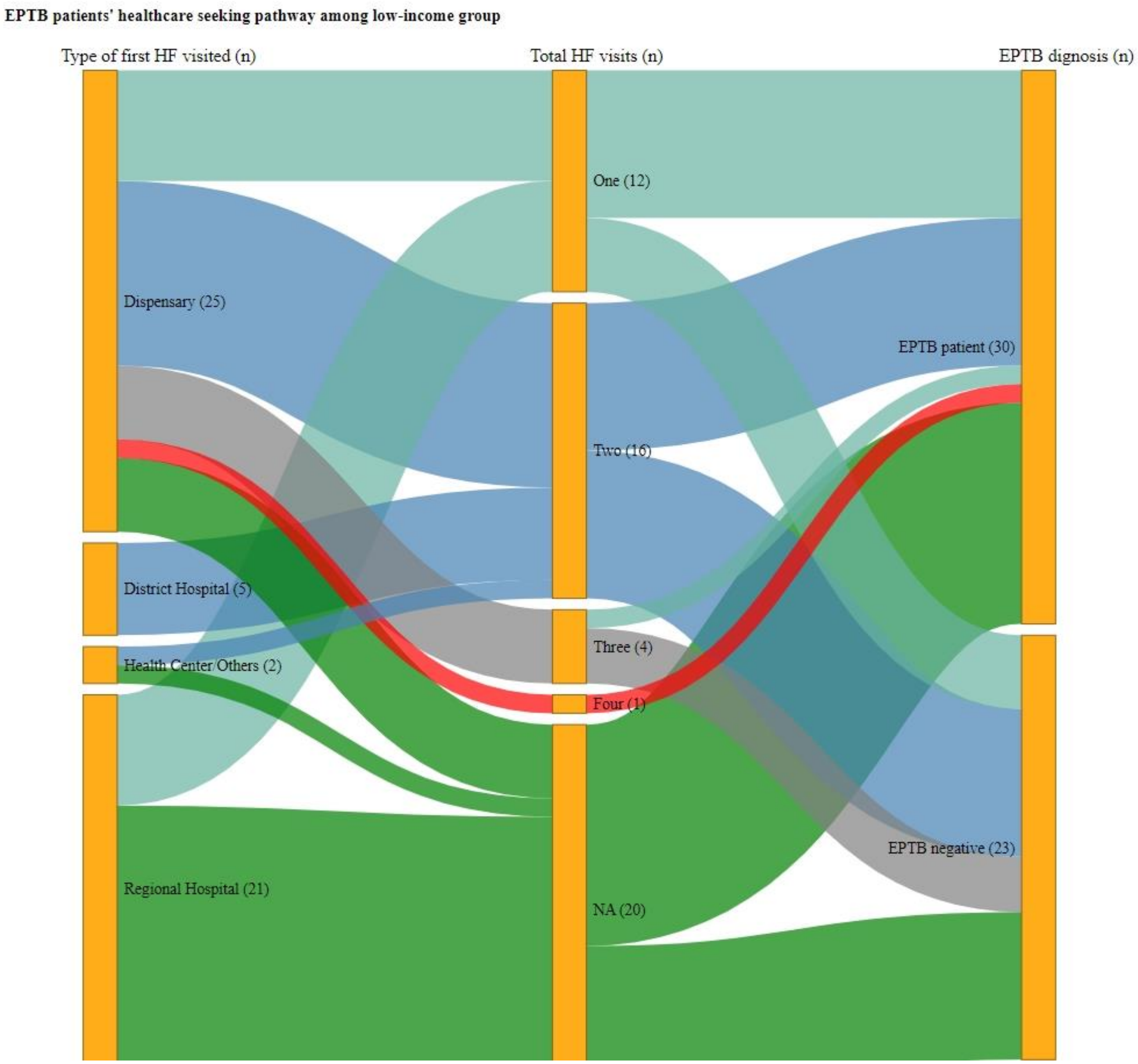

**Fig. D. Illustrations of healthcare access pathways followed by presumptive EPTB patients among middle-income group.**

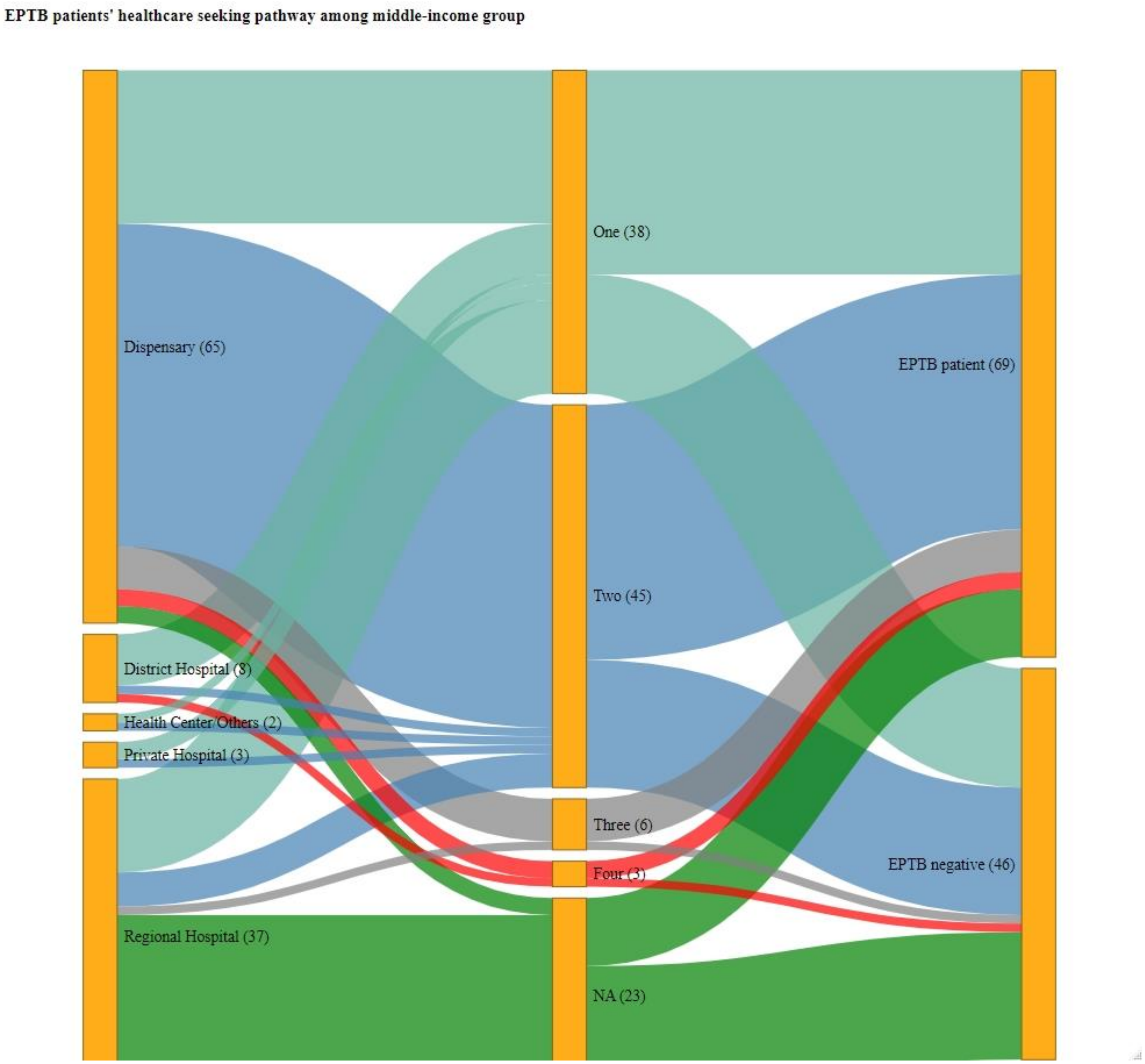

**Fig. E. Illustrations of healthcare access pathways followed by presumptive EPTB patients among high-income groups.**

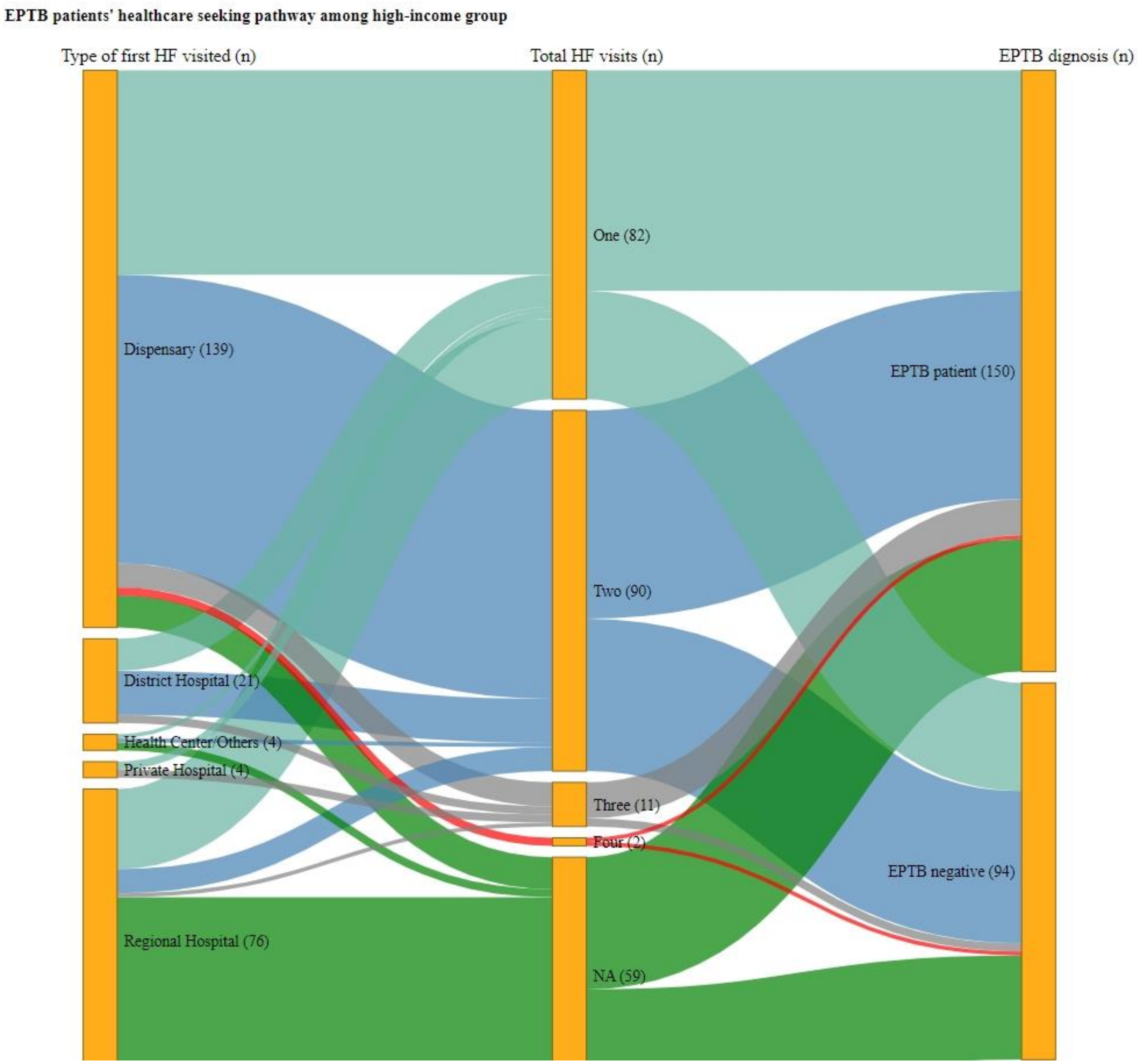

**Fig. F. Illustrations of healthcare access pathways followed by presumptive EPTB patients having lymphadenitis.**

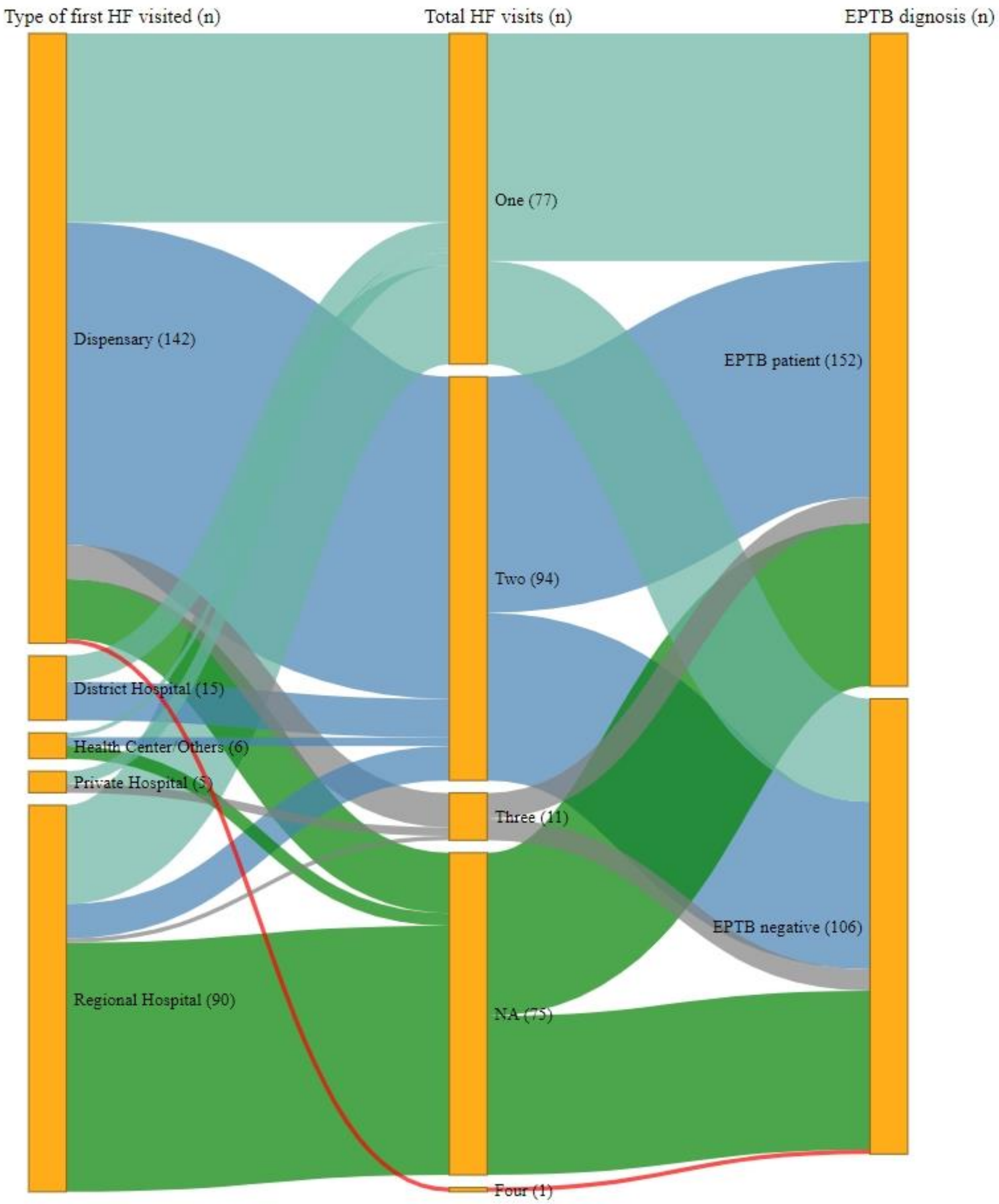

**Fig. G. Illustrations of healthcare access pathways followed by presumptive EPTB patients having pleuritis.**

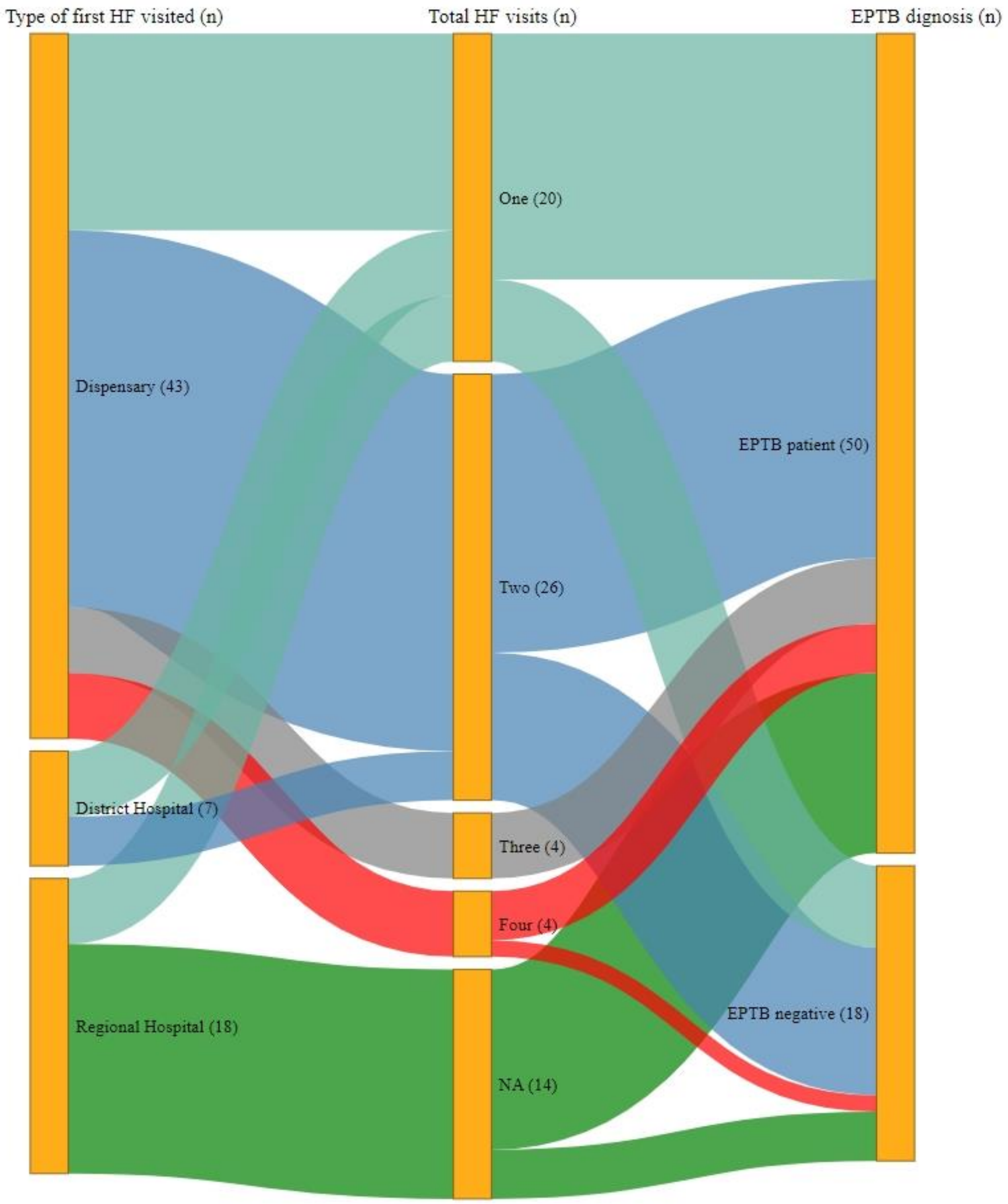

**Fig. H: Illustrations of healthcare access pathways followed by presumptive EPTB patients having meningitis.**

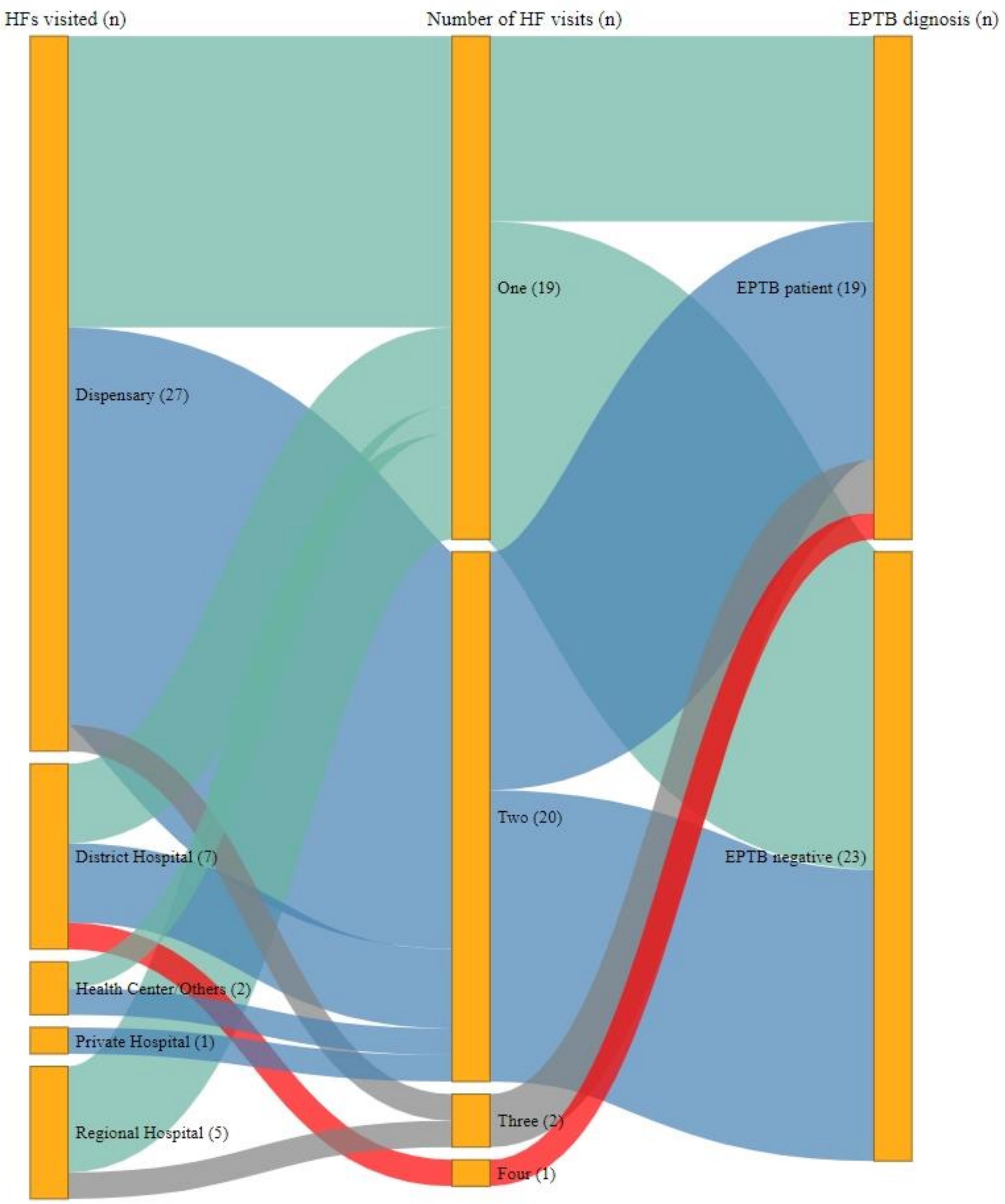

**S Table 1: Descriptive analysis of sociodemographic characteristics and individual-, societal-level and structural barriers in access to health care among presumptive EPTB patients based on site of infection.**

| | | | EPTB patients by affected body sites as disease manifestation | | | | | | Non-TB cases by affected body sites as disease manifestation | | | | | |
|---|---|---|---|---|---|---|---|---|---|---|---|---|---|---|
| | | | Lymphadenitis (%) | Pleuritis (%) | Meningitis (%) | Ascites (%) | Others (%) | p-value* | Lymphadenitis (%) | Pleuritis (%) | Meningitis (%) | Ascites (%) | Others (%) | p-value* |
| | Subgroups | Categories | 152 (61.0) | 50 (20.1) | 21 (8.4) | 8 (3.2) | 18 (7.2) | | 106 (65.0) | 18 (11.0) | 31 (19.0) | 1 (0.6) | 7 (4.3) | |
| Individual (patient-level) | Age Groups (years) | <14 | 20 (13.2) | 1 (2.0) | 1 (4.8) | 0 (0.0) | 1 (5.6) | <0.001 | 27 (25.5) | 1 (5.6) | 7 (22.6) | 0 (0.0) | 1 (14.3) | 0.018 |
| | | 15-24 | 57 (37.5) | 18 (36.0) | 6 (28.6) | 1 (12.5) | 3 (16.7) | | 24 (22.6) | 2 (11.1) | 1 (3.2) | 0 (0.0) | 0 (0.0) | |
| | | 25-44 | 59 (38.8) | 19 (38.0) | 7 (33.3) | 3 (37.5) | 7 (38.9) | | 33 (31.1) | 5 (27.8) | 6 (19.4) | 1 (100.0) | 2 (28.6) | |
| | | 45-64 | 15 (9.9) | 7 (14.0) | 1 (4.8) | 4 (50.0) | 5 (27.8) | | 16 (15.1) | 8 (44.4) | 10 (32.3) | 0 (0.0) | 3 (42.9) | |
| | | >=65 | 1 (0.7) | 5 (10.0) | 6 (28.6) | 0 (0.0) | 2 (11.1) | | 6 (5.7) | 2 (11.1) | 7 (22.6) | 0 (0.0) | 1 (14.3) | |
| | Gender | Female | 105 (69.1) | 13 (26.0) | 7 (33.3) | 5 (62.5) | 10 (55.6) | <0.001 | 47 (44.3) | 7 (38.9) | 14 (45.2) | 0 (0.0) | 4 (57.1) | 0.829 |
| | | Male | 47 (30.9) | 37 (74.0) | 14 (66.7) | 3 (37.5) | 8 (44.4) | | 59 (55.7) | 11 (61.1) | 17 (54.8) | 1 (100.0) | 3 (42.9) | |
| | Marital status | Married | 85 (63.9) | 38 (77.6) | 14 (73.7) | 6 (75.0) | 12 (70.6) | 0.464 | 52 (65.0) | 14 (77.8) | 22 (84.6) | 1 (100.0) | 6 (85.7) | 0.255 |
| | | Unmarried | 48 (36.1) | 11 (22.4) | 5 (26.3) | 2 (25.0) | 5 (29.4) | | 28 (35.0) | 4 (22.2) | 4 (15.4) | 0 (0.0) | 1 (14.3) | |
| | Education levels | Primary or below | 84 (55.3) | 32 (64.0) | 16 (76.2) | 7 (87.5) | 13 (72.2) | 0.039 | 70 (66.0) | 11 (61.1) | 24 (77.4) | 1 (100.0) | 5 (71.4) | 0.905 |
| | | Middle or Secondary | 46 (30.3) | 18 (36.0) | 4 (19.0) | 0 (0.0) | 4 (22.2) | | 27 (25.5) | 6 (33.3) | 5 (16.1) | 0 (0.0) | 2 (28.6) | |
| | | Higher | 22 (14.5) | 0 (0.0) | 1 (4.8) | 1 (12.5) | 1 (5.6) | | 9 (8.5) | 1 (5.6) | 2 (6.5) | 0 (0.0) | 0 (0.0) | |
| | Occupation | Govt Employed | 26 (17.1) | 12 (24.0) | 5 (23.8) | 3 (37.5) | 4 (22.2) | 0.041 | 25 (23.6) | 4 (22.2) | 7 (22.6) | 1 (100.0) | 2 (28.6) | 0.692 |
| | | Housewife | 57 (37.5) | 8 (16.0) | 2 (9.5) | 3 (37.5) | 5 (27.8) | | 27 (25.5) | 3 (16.7) | 7 (22.6) | 0 (0.0) | 3 (42.9) | |
| | | Unemployed | 37 (24.3) | 9 (18.0) | 5 (23.8) | 1 (12.5) | 3 (16.7) | | 29 (27.4) | 5 (27.8) | 5 (16.1) | 0 (0.0) | 1 (14.3) | |
| | | Private Employed | 32 (21.1) | 21 (42.0) | 9 (42.9) | 1 (12.5) | 6 (33.3) | | 25 (23.6) | 6 (33.3) | 12 (38.7) | 0 (0.0) | 1 (14.3) | |
| | Salary Categories | Low income | 21 (13.8) | 4 (8.0) | 3 (14.3) | 1 (12.5) | 1 (5.6) | 0.352 | 18 (17.0) | 3 (16.7) | 0 (0.0) | 1 (100.0) | 1 (14.3) | 0.073 |
| | | Middle income | 35 (23.0) | 19 (38.0) | 9 (42.9) | 2 (25.0) | 4 (22.2) | | 31 (29.2) | 3 (16.7) | 9 (29.0) | 0 (0.0) | 3 (42.9) | |
| | | High income | 96 (63.2) | 27 (54.0) | 9 (42.9) | 5 (62.5) | 13 (72.2) | | 57 (53.8) | 12 (66.7) | 22 (71.0) | 0 (0.0) | 3 (42.9) | |
| | Number of family members | 1-4 | 50 (32.9) | 16 (32.0) | 10 (47.6) | 1 (12.5) | 7 (38.9) | 0.766 | 31 (29.2) | 5 (27.8) | 11 (35.5) | 0 (0.0) | 1 (14.3) | 0.060 |
| | | 5-7 | 78 (51.3) | 22 (44.0) | 8 (38.1) | 5 (62.5) | 7 (38.9) | | 58 (54.7) | 10 (55.6) | 14 (45.2) | 1 (100.0) | 4 (57.1) | |
| | | 8-10 | 19 (12.5) | 10 (20.0) | 2 (9.5) | 2 (25.0) | 4 (22.2) | | 14 (13.2) | 3 (16.7) | 6 (19.4) | 0 (0.0) | 0 (0.0) | |
| | | 11-20 | 5 (3.3) | 2 (4.0) | 1 (4.8) | 0 (0.0) | 0 (0.0) | | 3 (2.8) | 0 (0.0) | 0 (0.0) | 0 (0.0) | 2 (28.6) | |
| | Had TB previously | No | 131 (86.2) | 49 (98.0) | 20 (95.2) | 8 (100.0) | 14 (77.8) | 0.047 | 98 (92.5) | 16 (88.9) | 30 (96.8) | 1 (100.0) | 7 (100.0) | 0.765 |
| | | Yes | 21 (13.8) | 1 (2.0) | 1 (4.8) | 0 (0.0) | 4 (22.2) | | 8 (7.5) | 2 (11.1) | 1 (3.2) | 0 (0.0) | 0 (0.0) | |
| | Family History of TB | No | 127 (83.6) | 42 (84.0) | 17 (81.0) | 8 (100.0) | 13 (72.2) | 0.514 | 98 (92.5) | 16 (88.9) | 29 (93.5) | 1 (100.0) | 6 (85.7) | 0.934 |
| | | Yes | 25 (16.4) | 8 (16.0) | 4 (19.0) | 0 (0.0) | 5 (27.8) | | 8 (7.5) | 2 (11.1) | 2 (6.5) | 0 (0.0) | 1 (14.3) | |
| | Heard of TB | No | 49 (32.2) | 7 (14.0) | 3 (14.3) | 1 (12.5) | 2 (11.1) | 0.023 | 40 (37.7) | 4 (22.2) | 11 (35.5) | 0 (0.0) | 3 (42.9) | 0.675 |
| | | Yes | 103 (67.8) | 43 (86.0) | 18 (85.7) | 7 (87.5) | 16 (88.9) | | 66 (62.3) | 14 (77.8) | 20 (64.5) | 1 (100.0) | 4 (57.1) | |
| | Self-medication for TB | No | 125 (82.2) | 39 (78.0) | 15 (71.4) | 4 (50.0) | 16 (88.9) | 0.140 | 92 (86.8) | 16 (88.9) | 19 (61.3) | 1 (100.0) | 7 (100.0) | 0.009 |
| | | Yes | 27 (17.8) | 11 (22.0) | 6 (28.6) | 4 (50.0) | 2 (11.1) | | 14 (13.2) | 2 (11.1) | 12 (38.7) | 0 (0.0) | 0 (0.0) | |
| Community (societal level) | Stigma associated with TB | Yes/Uncertain | 73 (48.0) | 28 (56.0) | 17 (81.0) | 6 (75.0) | 6 (33.3) | 0.014 | 61 (57.5) | 12 (66.7) | 26 (83.9) | 1 (100.0) | 2 (28.6) | 0.023 |
| | | No | 79 (52.0) | 22 (44.0) | 4 (19.0) | 2 (25.0) | 12 (66.7) | | 45 (42.5) | 6 (33.3) | 5 (16.1) | 0 (0.0) | 5 (71.4) | |
| Structural barriers | First HF visited for this illness | Dispensary | 78 (51.3) | 30 (60.0) | 12 (57.1) | 4 (50.0) | 10 (55.6) | 0.548 | 64 (60.4) | 13 (72.2) | 15 (48.4) | 1 (100.0) | 2 (28.6) | 0.581 |
| | | District Hospital | 12 (7.9) | 5 (10.0) | 3 (14.3) | 2 (25.0) | 2 (11.1) | | 3 (2.8) | 2 (11.1) | 4 (12.9) | 0 (0.0) | 1 (14.3) | |
| | | Health Center/Others | 5 (3.3) | 0 (0.0) | 1 (4.8) | 0 (0.0) | 0 (0.0) | | 1 (0.9) | 0 (0.0) | 1 (3.2) | 0 (0.0) | 0 (0.0) | |
| | | Private Hospital | 4 (2.6) | 0 (0.0) | 0 (0.0) | 1 (12.5) | 0 (0.0) | | 1 (0.9) | 0 (0.0) | 1 (3.2) | 0 (0.0) | 0 (0.0) | |
| | | Regional Hospital | 53 (34.9) | 15 (30.0) | 5 (23.8) | 1 (12.5) | 6 (33.3) | | 37 (34.9) | 3 (16.7) | 10 (32.3) | 0 (0.0) | 4 (57.1) | |
| | Number of HFs visited | One | 53 (34.9) | 15 (30.0) | 7 (33.3) | 5 (62.5) | 7 (38.9) | 0.104 | 24 (22.6) | 5 (27.8) | 12 (38.7) | 0 (0.0) | 4 (57.1) | 0.001 |
| | | Two | 55 (36.2) | 17 (34.0) | 9 (42.9) | 3 (37.5) | 6 (33.3) | | 39 (36.8) | 9 (50.0) | 11 (35.5) | 0 (0.0) | 2 (28.6) | |
| | | Three | 6 (3.9) | 4 (8.0) | 2 (9.5) | 0 (0.0) | 3 (16.7) | | 5 (4.7) | 0 (0.0) | 0 (0.0) | 1 (100.0) | 0 (0.0) | |
| | | Four | 0 (0.0) | 3 (6.0) | 1 (4.8) | 0 (0.0) | 0 (0.0) | | 1 (0.9) | 1 (5.6) | 0 (0.0) | 0 (0.0) | 0 (0.0) | |
| | | Missing | 38 (25.0) | 11 (22.0) | 2 (9.5) | 0 (0.0) | 2 (11.1) | | 37 (34.9) | 3 (16.7) | 8 (25.8) | 0 (0.0) | 1 (14.3) | |
| | Number of visits to HFs | One | 48 (31.6) | 13 (26.0) | 3 (14.3) | 1 (12.5) | 5 (27.8) | <0.001 | 24 (22.6) | 3 (16.7) | 7 (22.6) | 0 (0.0) | 4 (57.1) | 0.216 |
| | | Two | 61 (40.1) | 11 (22.0) | 12 (57.1) | 7 (87.5) | 7 (38.9) | | 33 (31.1) | 8 (44.4) | 13 (41.9) | 0 (0.0) | 2 (28.6) | |
| | | Three | 10 (6.6) | 9 (18.0) | 3 (14.3) | 0 (0.0) | 2 (11.1) | | 13 (12.3) | 5 (27.8) | 4 (12.9) | 1 (100.0) | 0 (0.0) | |
| | | Four | 2 (1.3) | 8 (16.0) | 2 (9.5) | 0 (0.0) | 3 (16.7) | | 4 (3.8) | 1 (5.6) | 1 (3.2) | 0 (0.0) | 0 (0.0) | |
| | | Missing | 31 (20.4) | 9 (18.0) | 1 (4.8) | 0 (0.0) | 1 (5.6) | | 32 (30.2) | 1 (5.6) | 6 (19.4) | 0 (0.0) | 1 (14.3) | |
| | Travel time to the nearest HF | Below 30 min | 62 (40.8) | 13 (26.0) | 7 (33.3) | 2 (25.0) | 5 (27.8) | 0.169 | 57 (53.8) | 5 (27.8) | 13 (41.9) | 0 (0.0) | 1 (14.3) | 0.177 |
| | | Between 30- 60 min | 66 (43.4) | 19 (38.0) | 9 (42.9) | 3 (37.5) | 8 (44.4) | | 36 (34.0) | 11 (61.1) | 12 (38.7) | 1 (100.0) | 5 (71.4) | |
| | | Above 60 min | 24 (15.8) | 18 (36.0) | 5 (23.8) | 3 (37.5) | 5 (27.8) | | 13 (12.3) | 2 (11.1) | 6 (19.4) | 0 (0.0) | 1 (14.3) | |
| | Travel time to this HF | Below 30 min | 78 (51.3) | 24 (48.0) | 8 (38.1) | 4 (50.0) | 9 (50.0) | 0.023 | 59 (55.7) | 11 (61.1) | 16 (51.6) | 0 (0.0) | 4 (57.1) | 0.263 |
| | | Between 30- 60 min | 48 (31.6) | 9 (18.0) | 5 (23.8) | 0 (0.0) | 2 (11.1) | | 24 (22.6) | 2 (11.1) | 3 (9.7) | 1 (100.0) | 1 (14.3) | |
| | | Above 60 min | 26 (17.1) | 17 (34.0) | 8 (38.1) | 4 (50.0) | 7 (38.9) | | 23 (21.7) | 5 (27.8) | 12 (38.7) | 0 (0.0) | 2 (28.6) | |
| | Travel & Wait time to this HF | Below 10 min | 114 (75.0) | 3 (6.0) | 0 (0.0) | 1 (12.5) | 1 (5.6) | <0.001 | 84 (79.2) | 3 (16.7) | 2 (6.5) | 0 (0.0) | 0 (0.0) | <0.001 |
| | | 10- 60 min | 27 (17.8) | 7 (14.0) | 3 (14.3) | 2 (25.0) | 5 (27.8) | | 21 (19.8) | 3 (16.7) | 6 (19.4) | 0 (0.0) | 3 (42.9) | |
| | | 60- 120 min | 8 (5.3) | 28 (56.0) | 10 (47.6) | 3 (37.5) | 6 (33.3) | | 0 (0.0) | 8 (44.4) | 18 (58.1) | 1 (100.0) | 2 (28.6) | |
| | | Above 120 min | 3 (2.0) | 12 (24.0) | 8 (38.1) | 2 (25.0) | 6 (33.3) | | 1 (0.9) | 4 (22.2) | 5 (16.1) | 0 (0.0) | 2 (28.6) | |

*p-value corresponds to the use of the Pearson chi-square test.

**S Table 2: Presumptive EPTB patients' characteristics among children based on sites of infection as disease manifestation.**

| | | | Lymphadenitis (%) | Pleuritis (%) | Meningitis (%) | Others (%) | p-value* |
|---|---|---|---|---|---|---|---|
| | Subgroups | Categories | 47 (79.7) | 2 (3.4) | 8 (13.6) | 2 (3.4) | |
| Individual (patient-level) | Gender | Female | 21 (44.7) | 1 (50.0) | 5 (62.5) | 2 (100.0) | 0.389 |
| | | Male | 26 (55.3) | 1 (50.0) | 3 (37.5) | 0 (0.0) | |
| | Education levels | Primary or below | 41 (87.2) | 2 (100.0) | 7 (87.5) | 2 (100.0) | 0.901 |
| | | Middle or Secondary | 6 (12.8) | 0 (0.0) | 1 (12.5) | 0 (0.0) | |
| | Occupation | Govt Employed | 18 (38.3) | 0 (0.0) | 1 (12.5) | 1 (50.0) | 0.362 |
| | | Housewife | 1 (2.1) | 0 (0.0) | 0 (0.0) | 0 (0.0) | |
| | | Unemployed | 11 (23.4) | 2 (100.0) | 2 (25.0) | 1 (50.0) | |
| | | Private Employed | 17 (36.2) | 0 (0.0) | 5 (62.5) | 0 (0.0) | |
| | Salary Categories | Low income | 6 (12.8) | 0 (0.0) | 0 (0.0) | 0 (0.0) | 0.929 |
| | | Middle income | 17 (36.2) | 1 (50.0) | 3 (37.5) | 1 (50.0) | |
| | | High income | 24 (51.1) | 1 (50.0) | 5 (62.5) | 1 (50.0) | |
| | Number of family members | 1-4 | 12 (25.5) | 0 (0.0) | 1 (12.5) | 1 (50.0) | 0.929 |
| | | 5-7 | 26 (55.3) | 2 (100.0) | 6 (75.0) | 1 (50.0) | |
| | | 8-10 | 7 (14.9) | 0 (0.0) | 1 (12.5) | 0 (0.0) | |
| | | 11-20 | 2 (4.3) | 0 (0.0) | 0 (0.0) | 0 (0.0) | |
| | Had TB previously | No | 44 (93.6) | 2 (100.0) | 8 (100.0) | 2 (100.0) | 0.848 |
| | | Yes | 3 (6.4) | 0 (0.0) | 0 (0.0) | 0 (0.0) | |
| | Family History of TB | No | 42 (89.4) | 2 (100.0) | 7 (87.5) | 1 (50.0) | 0.373 |
| | | Yes | 5 (10.6) | 0 (0.0) | 1 (12.5) | 1 (50.0) | |
| | Heard of TB | No | 18 (38.3) | 0 (0.0) | 3 (37.5) | 1 (50.0) | 0.718 |
| | | Yes | 29 (61.7) | 2 (100.0) | 5 (62.5) | 1 (50.0) | |
| | Self-medication for TB | No | 39 (83.0) | 2 (100.0) | 5 (62.5) | 2 (100.0) | 0.412 |
| | | Yes | 8 (17.0) | 0 (0.0) | 3 (37.5) | 0 (0.0) | |
| Community (societal level) | Stigma associated with TB | Yes/Uncertain | 24 (51.1) | 2 (100.0) | 7 (87.5) | 0 (0.0) | 0.050 |
| | | No | 23 (48.9) | 0 (0.0) | 1 (12.5) | 2 (100.0) | |
| Structural barriers | Something can be done | Yes | 0 (0.0) | 0 (0.0) | 0 (0.0) | 0 (0.0) | 0.244 |
| | | No | 41 (87.2) | 2 (100.0) | 8 (100.0) | 1 (50.0) | |
| | | Uncertain | 6 (12.8) | 0 (0.0) | 0 (0.0) | 1 (50.0) | |
| | First HF visited for this illness | Dispensary | 31 (66.0) | 2 (100.0) | 3 (37.5) | 0 (0.0) | 0.108 |
| | | District Hospital | 1 (2.1) | 0 (0.0) | 2 (25.0) | 0 (0.0) | |
| | | Health Center/Others | 0 (0.0) | 0 (0.0) | 0 (0.0) | 0 (0.0) | |
| | | Private Hospital | 2 (4.3) | 0 (0.0) | 0 (0.0) | 0 (0.0) | |
| | | Regional Hospital | 13 (27.7) | 0 (0.0) | 3 (37.5) | 2 (100.0) | |
| | Number of HFs visited | One | 14 (29.8) | 0 (0.0) | 2 (25.0) | 0 (0.0) | 0.002 |
| | | Two | 20 (42.6) | 1 (50.0) | 4 (50.0) | 1 (50.0) | |
| | | Three | 2 (4.3) | 0 (0.0) | 0 (0.0) | 0 (0.0) | |
| | | Four | 0 (0.0) | 1 (50.0) | 0 (0.0) | 0 (0.0) | |
| | | Missing | 11 (23.4) | 0 (0.0) | 2 (25.0) | 1 (50.0) | |
| | Times of visits | One | 13 (27.7) | 0 (0.0) | 2 (25.0) | 0 (0.0) | 0.293 |
| | | Two | 19 (40.4) | 1 (50.0) | 3 (37.5) | 1 (50.0) | |
| | | Three | 6 (12.8) | 0 (0.0) | 0 (0.0) | 0 (0.0) | |
| | | Four | 1 (2.1) | 1 (50.0) | 1 (12.5) | 0 (0.0) | |
| | | Missing | 8 (17.0) | 0 (0.0) | 2 (25.0) | 1 (50.0) | |
| | Travel time to the nearest HF | Below 30 min | 26 (55.3) | 0 (0.0) | 0 (0.0) | 0 (0.0) | 0.058 |
| | | Between 30- 60 min | 12 (25.5) | 1 (50.0) | 5 (62.5) | 1 (50.0) | |
| | | Above 60 min | 9 (19.1) | 1 (50.0) | 3 (37.5) | 1 (50.0) | |
| | Travel time to this HF | Below 30 min | 25 (53.2) | 0 (0.0) | 4 (50.0) | 1 (50.0) | 0.665 |
| | | Between 30- 60 min | 14 (29.8) | 1 (50.0) | 2 (25.0) | 0 (0.0) | |
| | | Above 60 min | 8 (17.0) | 1 (50.0) | 2 (25.0) | 1 (50.0) | |
| | Travel & Wait time to this HF | Below 10 min | 38 (80.9) | 1 (50.0) | 1 (12.5) | 0 (0.0) | <0.001 |
| | | 10- 60 min | 8 (17.0) | 1 (50.0) | 2 (25.0) | 1 (50.0) | |
| | | 60- 120 min | 0 (0.0) | 0 (0.0) | 5 (62.5) | 1 (50.0) | |
| | | Above 120 min | 1 (2.1) | 0 (0.0) | 0 (0.0) | 0 (0.0) | |

* p-value corresponds to the use of the Pearson chi-square test.

**S Table 3: Health facilities accessed by subgroups of presumptive EPTB patients for their first access to healthcare.**

| | | Presumptive EPTB patients' HF visits | | | | | |
|---|---|---|---|---|---|---|---|
| | | Dispensary (%) | District Hospital (%) | Health Center/Others (%) | Private Hospital (%) | Regional Hospital (%) | p-value* |
| Subgroups | Categories | 229 (55.6) | 34 (8.3) | 8 (1.9) | 7 (1.7) | 134 (32.5) | |
| Gender | Female | 116 (50.7) | 14 (41.2) | 4 (50.0) | 4 (57.1) | 74 (55.2) | 0.671 |
| | Male | 113 (49.3) | 20 (58.8) | 4 (50.0) | 3 (42.9) | 60 (44.8) | |
| Salary Categories | Low income | 25 (10.9) | 5 (14.7) | 2 (25.0) | 0 (0.0) | 21 (15.7) | 0.797 |
| | Middle income | 65 (28.4) | 8 (23.5) | 2 (25.0) | 3 (42.9) | 37 (27.6) | |
| | High income | 139 (60.7) | 21 (61.8) | 4 (50.0) | 4 (57.1) | 76 (56.7) | |
| Affected body site | Lymphadenitis | 142 (62.0) | 15 (44.1) | 6 (75.0) | 5 (71.4) | 90 (67.2) | 0.21 |
| | Pleuritis | 43 (18.8) | 7 (20.6) | 0 (0.0) | 0 (0.0) | 18 (13.4) | |
| | Meningitis | 27 (11.8) | 7 (20.6) | 2 (25.0) | 1 (14.3) | 15 (11.2) | |
| | Ascites | 5 (2.2) | 2 (5.9) | 0 (0.0) | 1 (14.3) | 1 (0.7) | |
| | Others | 12 (5.2) | 3 (8.8) | 0 (0.0) | 0 (0.0) | 10 (7.5) | |

*p-value corresponds to the use of the Pearson chi-square test.

**S Table 4: Subgroup analysis of presumptive EPTB patients' access to health facilities against the total number of visits.**

| Subgroups | Categories | | | One | Two | Three | Four | NA | p-value* |
|---|---|---|---|---|---|---|---|---|---|
| Gender | Female | Total N (%) | | 54 (25.5) | 78 (36.8) | 23 (10.8) | 9 (4.2) | 48 (22.6) | |
| | | First HF visited for this illness | Dispensary | 24 (44.4) | 57 (73.1) | 19 (82.6) | 7 (77.8) | 9 (18.8) | <0.001 |
| | | | District Hospital | 2 (3.7) | 9 (11.5) | 1 (4.3) | 2 (22.2) | 0 (0.0) | |
| | | | Health Center/Others | 0 (0.0) | 2 (2.6) | 0 (0.0) | 0 (0.0) | 2 (4.2) | |
| | | | Private Hospital | 2 (3.7) | 1 (1.3) | 1 (4.3) | 0 (0.0) | 0 (0.0) | |
| | | | Regional Hospital | 26 (48.1) | 9 (11.5) | 2 (8.7) | 0 (0.0) | 37 (77.1) | |
| | | EPTB Diagnosis | EPTB patient | 36 (66.7) | 55 (70.5) | 13 (56.5) | 7 (77.8) | 29 (60.4) | 0.581 |
| | | | EPTB negative | 18 (33.3) | 23 (29.5) | 10 (43.5) | 2 (22.2) | 19 (39.6) | |
| | Male | Total N (%) | | 54 (27.0) | 76 (38.0) | 24 (12.0) | 12 (6.0) | 34 (17.0) | |
| | | First HF visited for this illness | Dispensary | 25 (46.3) | 52 (68.4) | 21 (87.5) | 10 (83.3) | 5 (14.7) | <0.001 |
| | | | District Hospital | 6 (11.1) | 11 (14.5) | 1 (4.2) | 2 (16.7) | 0 (0.0) | |
| | | | Health Center/Others | 0 (0.0) | 3 (3.9) | 0 (0.0) | 0 (0.0) | 1 (2.9) | |
| | | | Private Hospital | 0 (0.0) | 2 (2.6) | 1 (4.2) | 0 (0.0) | 0 (0.0) | |
| | | | Regional Hospital | 23 (42.6) | 8 (10.5) | 1 (4.2) | 0 (0.0) | 28 (82.4) | |
| | | EPTB Diagnosis | EPTB patient | 34 (63.0) | 43 (56.6) | 11 (45.8) | 8 (66.7) | 13 (38.2) | 0.149 |
| | | | EPTB negative | 20 (37.0) | 33 (43.4) | 13 (54.2) | 4 (33.3) | 21 (61.8) | |
| Affordability status | Low income | Total N (%) | | 15 (28.3) | 12 (22.6) | 6 (11.3) | 4 (7.5) | 16 (30.2) | |
| | | First HF visited for this illness | Dispensary | 6 (40.0) | 7 (58.3) | 5 (83.3) | 3 (75.0) | 4 (25.0) | 0.012 |
| | | | District Hospital | 0 (0.0) | 3 (25.0) | 1 (16.7) | 1 (25.0) | 0 (0.0) | |
| | | | Health Center/Others | 0 (0.0) | 1 (8.3) | 0 (0.0) | 0 (0.0) | 1 (6.2) | |
| | | | Private Hospital | 0 (0.0) | 0 (0.0) | 0 (0.0) | 0 (0.0) | 0 (0.0) | |
| | | | Regional Hospital | 9 (60.0) | 1 (8.3) | 0 (0.0) | 0 (0.0) | 11 (68.8) | |
| | | EPTB Diagnosis | EPTB patient | 8 (53.3) | 6 (50.0) | 3 (50.0) | 3 (75.0) | 10 (62.5) | 0.884 |
| | | | EPTB negative | 7 (46.7) | 6 (50.0) | 3 (50.0) | 1 (25.0) | 6 (37.5) | |
| | Middle income | Total N (%) | | 28 (24.3) | 47 (40.9) | 14 (12.2) | 8 (7.0) | 18 (15.7) | |
| | | First HF visited for this illness | Dispensary | 13 (46.4) | 30 (63.8) | 13 (92.9) | 7 (87.5) | 2 (11.1) | <0.001 |
| | | | District Hospital | 3 (10.7) | 4 (8.5) | 0 (0.0) | 1 (12.5) | 0 (0.0) | |
| | | | Health Center/Others | 0 (0.0) | 2 (4.3) | 0 (0.0) | 0 (0.0) | 0 (0.0) | |
| | | | Private Hospital | 1 (3.6) | 2 (4.3) | 0 (0.0) | 0 (0.0) | 0 (0.0) | |
| | | | Regional Hospital | 11 (39.3) | 9 (19.1) | 1 (7.1) | 0 (0.0) | 16 (88.9) | |
| | | EPTB Diagnosis | EPTB patient | 17 (60.7) | 29 (61.7) | 11 (78.6) | 6 (75.0) | 6 (33.3) | 0.086 |
| | | | EPTB negative | 11 (39.3) | 18 (38.3) | 3 (21.4) | 2 (25.0) | 12 (66.7) | |
| | High income | Total N (%) | | 65 (26.6) | 95 (38.9) | 27 (11.1) | 9 (3.7) | 48 (19.7) | |
| | | First HF visited for this illness | Dispensary | 30 (46.2) | 72 (75.8) | 22 (81.5) | 7 (77.8) | 8 (16.7) | <0.001 |
| | | | District Hospital | 5 (7.7) | 13 (13.7) | 1 (3.7) | 2 (22.2) | 0 (0.0) | |
| | | | Health Center/Others | 0 (0.0) | 2 (2.1) | 0 (0.0) | 0 (0.0) | 2 (4.2) | |
| | | | Private Hospital | 1 (1.5) | 1 (1.1) | 2 (7.4) | 0 (0.0) | 0 (0.0) | |
| | | | Regional Hospital | 29 (44.6) | 7 (7.4) | 2 (7.4) | 0 (0.0) | 38 (79.2) | |
| | | EPTB Diagnosis | EPTB patient | 45 (69.2) | 63 (66.3) | 10 (37.0) | 6 (66.7) | 26 (54.2) | 0.032 |
| | | | EPTB negative | 20 (30.8) | 32 (33.7) | 17 (63.0) | 3 (33.3) | 22 (45.8) | |
| Site of infection | Lymphadenitis | Total N (%) | | 72 (27.9) | 94 (36.4) | 23 (8.9) | 6 (2.3) | 63 (24.4) | |
| | | First HF visited for this illness | Dispensary | 34 (47.2) | 71 (75.5) | 17 (73.9) | 6 (100.0) | 14 (22.2) | <0.001 |
| | | | District Hospital | 4 (5.6) | 10 (10.6) | 1 (4.3) | 0 (0.0) | 0 (0.0) | |
| | | | Health Center/Others | 0 (0.0) | 3 (3.2) | 0 (0.0) | 0 (0.0) | 3 (4.8) | |
| | | | Private Hospital | 2 (2.8) | 1 (1.1) | 2 (8.7) | 0 (0.0) | 0 (0.0) | |
| | | | Regional Hospital | 32 (44.4) | 9 (9.6) | 3 (13.0) | 0 (0.0) | 46 (73.0) | |
| | | EPTB Diagnosis | EPTB patient | 48 (66.7) | 61 (64.9) | 10 (43.5) | 2 (33.3) | 31 (49.2) | 0.049 |
| | | | EPTB negative | 24 (33.3) | 33 (35.1) | 13 (56.5) | 4 (66.7) | 32 (50.8) | |
| | Pleuritis | Total N (%) | | 16 (23.5) | 19 (27.9) | 14 (20.6) | 9 (13.2) | 10 (14.7) | |
| | | First HF visited for this illness | Dispensary | 8 (50.0) | 13 (68.4) | 13 (92.9) | 9 (100.0) | 0 (0.0) | <0.001 |
| | | | District Hospital | 2 (12.5) | 4 (21.1) | 1 (7.1) | 0 (0.0) | 0 (0.0) | |
| | | | Health Center/Others | 0 (0.0) | 0 (0.0) | 0 (0.0) | 0 (0.0) | 0 (0.0) | |
| | | | Private Hospital | 0 (0.0) | 0 (0.0) | 0 (0.0) | 0 (0.0) | 0 (0.0) | |
| | | | Regional Hospital | 6 (37.5) | 2 (10.5) | 0 (0.0) | 0 (0.0) | 10 (100.0) | |
| | | EPTB Diagnosis | EPTB patient | 13 (81.2) | 11 (57.9) | 9 (64.3) | 8 (88.9) | 9 (90.0) | 0.201 |
| | | | EPTB negative | 3 (18.8) | 8 (42.1) | 5 (35.7) | 1 (11.1) | 1 (10.0) | |
| | Meningitis | Total N (%) | | 10 (19.2) | 25 (48.1) | 7 (13.5) | 3 (5.8) | 7 (13.5) | |
| | | First HF visited for this illness | Dispensary | 3 (30.0) | 16 (64.0) | 7 (100.0) | 1 (33.3) | 0 (0.0) | 0.001 |
| | | | District Hospital | 2 (20.0) | 3 (12.0) | 0 (0.0) | 2 (66.7) | 0 (0.0) | |
| | | | Health Center/Others | 0 (0.0) | 2 (8.0) | 0 (0.0) | 0 (0.0) | 0 (0.0) | |
| | | | Private Hospital | 0 (0.0) | 1 (4.0) | 0 (0.0) | 0 (0.0) | 0 (0.0) | |

| | | | | | | | | | |
|---|---|---|---|---|---|---|---|---|---|
| | | | Regional Hospital | 5 (50.0) | 3 (12.0) | 0 (0.0) | 0 (0.0) | 7 (100.0) | |
| | | EPTB Diagnosis | EPTB patient | 3 (30.0) | 12 (48.0) | 3 (42.9) | 2 (66.7) | 1 (14.3) | 0.418 |
| | | | EPTB negative | 7 (70.0) | 13 (52.0) | 4 (57.1) | 1 (33.3) | 6 (85.7) | |

*p-value corresponds to the use of the Pearson chi-square test.

**S Table 5: Subgroup analysis of confirmed EPTB patients' total number of HF visits.**

| | | One | Two | Three | Four | NA | p-value* |
|---|---|---|---|---|---|---|---|
| Subgroups | Categories | 70 (28.1) | 98 (39.4) | 24 (9.6) | 15 (6.0) | 42 (16.9) | |
| Gender | Female | 36 (51.4) | 55 (56.1) | 13 (54.2) | 7 (46.7) | 29 (69.0) | 0.398 |
| | Male | 34 (48.6) | 43 (43.9) | 11 (45.8) | 8 (53.3) | 13 (31.0) | |
| Salary Categories | Low income | 8 (11.4) | 6 (6.1) | 3 (12.5) | 3 (20.0) | 10 (23.8) | 0.020 |
| | Middle income | 17 (24.3) | 29 (29.6) | 11 (45.8) | 6 (40.0) | 6 (14.3) | |
| | High income | 45 (64.3) | 63 (64.3) | 10 (41.7) | 6 (40.0) | 26 (61.9) | |
| Affected body site | Lymphadenitis | 48 (68.6) | 61 (62.2) | 10 (41.7) | 2 (13.3) | 31 (73.8) | <0.001 |
| | Pleuritis | 13 (18.6) | 11 (11.2) | 9 (37.5) | 8 (53.3) | 9 (21.4) | |
| | Meningitis | 3 (4.3) | 12 (12.2) | 3 (12.5) | 2 (13.3) | 1 (2.4) | |
| | Ascites | 1 (1.4) | 7 (7.1) | 0 (0.0) | 0 (0.0) | 0 (0.0) | |
| | Others | 5 (7.1) | 7 (7.1) | 2 (8.3) | 3 (20.0) | 1 (2.4) | |

*p-value corresponds to the use of the Pearson chi-square test.

**S Table 6: A descriptive analysis of EPTB patients' first HF visit against referral to the study site that provided EPTB diagnostic confirmation.**

| | | Referring entity to the health facility (study site) that provided the diagnosis | | | | | |
|---|---|---|---|---|---|---|---|
| | | Private facility | Government HF | Member of the family | Self | Other | p-value* |
| | **Total N (%)** | 4 (5.7) | 1 (1.4) | 4 (5.7) | 58 (82.9) | 3 (4.3) | |
| First HF visited among EPTB patients | Dispensary | 4 (100.0) | 0 (0.0) | 1 (25.0) | 24 (41.4) | 2 (66.7) | 0.002 |
| | District Hospital | 0 (0.0) | 1 (100.0) | 2 (50.0) | 2 (3.4) | 0 (0.0) | |
| | Health Center/Others | 0 (0.0) | 0 (0.0) | 0 (0.0) | 0 (0.0) | 0 (0.0) | |
| | Private Hospital | 0 (0.0) | 0 (0.0) | 0 (0.0) | 2 (3.4) | 0 (0.0) | |
| | Regional Hospital | 0 (0.0) | 0 (0.0) | 1 (25.0) | 30 (51.7) | 1 (33.3) | |
| First HF visited among non-TB patients | Dispensary | 4 (66.7) | 0 (0.0) | 1 (100.0) | 12 (41.4) | 1 (100.0) | 0.049 |
| | District Hospital | 0 (0.0) | 1 (100.0) | 0 (0.0) | 2 (6.9) | 0 (0.0) | |
| | Health Center/Others | 0 (0.0) | 0 (0.0) | 0 (0.0) | 0 (0.0) | 0 (0.0) | |
| | Private Hospital | 0 (0.0) | 0 (0.0) | 0 (0.0) | 0 (0.0) | 0 (0.0) | |
| | Regional Hospital | 2 (33.3) | 0 (0.0) | 0 (0.0) | 15 (51.7) | 0 (0.0) | |
| First HF visited among children group of presumptive EPTB patients | Dispensary | 1 (50.0) | 0 (0.0) | 1 (100.0) | 5 (45.5) | 0 (NaN) | 0.059 |
| | District Hospital | 0 (0.0) | 1 (100.0) | 0 (0.0) | 0 (0.0) | 0 (NaN) | |
| | Health Center/Others | 0 (0.0) | 0 (0.0) | 0 (0.0) | 0 (0.0) | 0 (NaN) | |
| | Private Hospital | 0 (0.0) | 0 (0.0) | 0 (0.0) | 1 (9.1) | 0 (NaN) | |
| | Regional Hospital | 1 (50.0) | 0 (0.0) | 0 (0.0) | 5 (45.5) | 0 (NaN) | |

*p-value corresponds to the use of the Pearson chi-square test.

**S Table 7: Factors associated with prolonged time to diagnosis among confirmed EPTB adult patients.**

| | Subgroups | Categories | By Patient Delay among confirmed EPTB patients | | | | By Health System Delay Phase I among confirmed EPTB patients | | | | By Health System Delay Phase II among confirmed EPTB patients | | | |
|---|---|---|---|---|---|---|---|---|---|---|---|---|---|---|
| | | | Below Median, n (%) | Above Median, n (%) | OR (Univariable, p-value) | OR (Multivariable, p-value) | Below Median, n (%) | Above Median, n (%) | OR (Univariable, p-value) | OR (Multivariable, p-value) | Below Median, n (%) | Above Median, n (%) | OR (Univariable, p-value) | OR (Multivariable, p-value) |
| Individual (patient-level) | Site of infection (EPTB manifestations) | Lymphadenitis | 64 (48.5) | 68 (51.5) | - | - | 59 (44.7) | 73 (55.3) | - | - | 86 (65.2) | 46 (34.8) | - | - |
| | | Pleuritis | 38 (77.6) | 11 (22.4) | 0.27 (0.12-0.56, p=0.001) | 0.24 (0.06-0.86, p=0.030) | 27 (55.1) | 22 (44.9) | 0.66 (0.34-1.27, p=0.214) | 0.97 (0.23-4.10, p=0.971) | 25 (51.0) | 24 (49.0) | 1.79 (0.92-3.50, p=0.085) | 10.13 (2.53-46.06, p=0.002) |
| | | Meningitis | 13 (65.0) | 7 (35.0) | 0.51 (0.18-1.32, p=0.174) | 0.82 (0.15-4.24, p=0.815) | 9 (45.0) | 11 (55.0) | 0.99 (0.38-2.60, p=0.980) | 1.98 (0.30-13.73, p=0.478) | 10 (50.0) | 10 (50.0) | 1.87 (0.72-4.88, p=0.195) | 15.18 (2.73-95.20, p=0.003) |
| | | Ascites | 5 (62.5) | 3 (37.5) | 0.56 (0.11-2.40, p=0.447) | 1.51 (0.17-12.03, p=0.700) | 4 (50.0) | 4 (50.0) | 0.81 (0.18-3.55, p=0.770) | 1.70 (0.19-15.87, p=0.636) | 6 (75.0) | 2 (25.0) | 0.62 (0.09-2.83, p=0.572) | 5.00 (0.35-61.68, p=0.213) |
| | | Others | 7 (41.2) | 10 (58.8) | 1.34 (0.49-3.90, p=0.571) | 3.17 (0.66-16.40, p=0.157) | 4 (23.5) | 13 (76.5) | 2.63 (0.88-9.71, p=0.106) | 12.61 (1.46-159.37, p=0.033) | 10 (62.5) | 6 (37.5) | 1.12 (0.36-3.22, p=0.834) | 4.47 (0.82-24.90, p=0.081) |
| | Age groups | 15-24 | 46 (54.1) | 39 (45.9) | - | - | 37 (43.5) | 48 (56.5) | - | - | 50 (58.8) | 35 (41.2) | - | - |
| | | 25-44 | 49 (51.6) | 46 (48.4) | 1.11 (0.62-1.99, p=0.733) | 0.99 (0.41-2.41, p=0.977) | 39 (41.1) | 56 (58.9) | 1.11 (0.61-2.00, p=0.737) | 0.63 (0.22-1.74, p=0.381) | 53 (56.4) | 41 (43.6) | 1.11 (0.61-2.01, p=0.742) | 0.77 (0.32-1.84, p=0.552) |
| | | 45-64 | 21 (65.6) | 11 (34.4) | 0.62 (0.26-1.42, p=0.264) | 0.51 (0.14-1.89, p=0.320) | 18 (56.2) | 14 (43.8) | 0.60 (0.26-1.36, p=0.221) | 0.15 (0.03-0.66, p=0.015) | 24 (75.0) | 8 (25.0) | 0.48 (0.18-1.15, p=0.110) | 0.21 (0.05-0.79, p=0.025) |
| | | >=65 | 11 (78.6) | 3 (21.4) | 0.32 (0.07-1.12, p=0.099) | 0.41 (0.05-2.69, p=0.367) | 9 (64.3) | 5 (35.7) | 0.43 (0.12-1.35, p=0.157) | 0.05 (0.01-0.42, p=0.007) | 10 (71.4) | 4 (28.6) | 0.57 (0.15-1.86, p=0.375) | 0.28 (0.04-1.63, p=0.167) |
| | Gender | Female | 60 (48.0) | 65 (52.0) | - | - | 53 (42.4) | 72 (57.6) | - | - | 77 (62.1) | 47 (37.9) | - | - |
| | | Male | 67 (66.3) | 34 (33.7) | 0.47 (0.27-0.80, p=0.006) | 1.19 (0.46-3.16, p=0.725) | 50 (49.5) | 51 (50.5) | 0.75 (0.44-1.27, p=0.287) | 0.47 (0.14-1.53, p=0.217) | 60 (59.4) | 41 (40.6) | 1.12 (0.65-1.92, p=0.681) | 0.89 (0.34-2.37, p=0.806) |
| | Marital Status | Married | 88 (56.8) | 67 (43.2) | - | - | 65 (41.9) | 90 (58.1) | - | - | 94 (61.0) | 60 (39.0) | - | - |
| | | Unmarried | 38 (54.3) | 32 (45.7) | 1.11 (0.63-1.95, p=0.728) | 1.50 (0.52-4.56, p=0.460) | 38 (54.3) | 32 (45.7) | 0.61 (0.34-1.07, p=0.086) | 0.22 (0.06-0.72, p=0.015) | 43 (61.4) | 27 (38.6) | 0.98 (0.55-1.75, p=0.956) | 1.06 (0.38-3.06, p=0.906) |
| | Education levels | Primary or below | 80 (59.7) | 54 (40.3) | - | - | 59 (44.0) | 75 (56.0) | - | - | 78 (58.6) | 55 (41.4) | - | - |
| | | Middle or Secondary | 32 (47.8) | 35 (52.2) | 1.62 (0.90-2.94, p=0.109) | 1.55 (0.68-3.54, p=0.296) | 32 (47.8) | 35 (52.2) | 0.86 (0.48-1.55, p=0.616) | 0.82 (0.32-2.13, p=0.687) | 43 (64.2) | 24 (35.8) | 0.79 (0.43-1.45, p=0.450) | 0.84 (0.37-1.89, p=0.681) |
| | | Higher | 15 (60.0) | 10 (40.0) | 0.99 (0.40-2.34, p=0.978) | 1.37 (0.42-4.49, p=0.596) | 12 (48.0) | 13 (52.0) | 0.85 (0.36-2.03, p=0.714) | 0.71 (0.18-2.85, p=0.627) | 16 (64.0) | 9 (36.0) | 0.80 (0.32-1.90, p=0.617) | 1.60 (0.45-5.58, p=0.461) |
| | Occupation | Govt Employed | 30 (68.2) | 14 (31.8) | - | - | 19 (43.2) | 25 (56.8) | - | - | 25 (56.8) | 19 (43.2) | - | - |
| | | Housewife | 30 (40.5) | 44 (59.5) | 3.14 (1.45-7.05, p=0.004) | 3.93 (1.14-14.38, p=0.034) | 30 (40.5) | 44 (59.5) | 1.11 (0.52-2.37, p=0.778) | 0.78 (0.18-3.30, p=0.737) | 47 (64.4) | 26 (35.6) | 0.73 (0.34-1.57, p=0.416) | 0.73 (0.21-2.54, p=0.623) |
| | | Unemployed | 30 (61.2) | 19 (38.8) | 1.36 (0.58-3.23, p=0.484) | 0.61 (0.16-2.22, p=0.463) | 28 (57.1) | 21 (42.9) | 0.57 (0.25-1.29, p=0.180) | 1.39 (0.34-5.90, p=0.646) | 36 (73.5) | 13 (26.5) | 0.48 (0.20-1.13, p=0.094) | 0.29 (0.08-1.00, p=0.054) |
| | | Private Employed | 37 (62.7) | 22 (37.3) | 1.27 (0.56-2.95, p=0.565) | 1.18 (0.38-3.70, p=0.777) | 26 (44.1) | 33 (55.9) | 0.96 (0.44-2.12, p=0.929) | 2.54 (0.78-8.67, p=0.127) | 29 (49.2) | 30 (50.8) | 1.36 (0.62-3.01, p=0.441) | 1.87 (0.67-5.37, p=0.236) |
| | Salary Categories | Low income | 9 (33.3) | 18 (66.7) | - | - | 17 (63.0) | 10 (37.0) | - | - | 16 (59.3) | 11 (40.7) | - | - |
| | | Middle income | 32 (52.5) | 29 (47.5) | 0.45 (0.17-1.14, p=0.101) | 0.72 (0.19-2.65, p=0.625) | 25 (41.0) | 36 (59.0) | 2.45 (0.98-6.40, p=0.060) | 1.19 (0.28-5.09, p=0.813) | 43 (70.5) | 18 (29.5) | 0.61 (0.24-1.58, p=0.303) | 0.73 (0.21-2.50, p=0.609) |
| | | High income | 86 (62.3) | 52 (37.7) | 0.30 (0.12-0.71, p=0.007) | 0.23 (0.07-0.75, p=0.018) | 61 (44.2) | 77 (55.8) | 2.15 (0.93-5.18, p=0.078) | 2.08 (0.54-8.09, p=0.286) | 78 (56.9) | 59 (43.1) | 1.10 (0.48-2.60, p=0.823) | 1.64 (0.57-4.94, p=0.367) |
| | Number of family members | 1-4 | 40 (50.6) | 39 (49.4) | - | - | 31 (39.2) | 48 (60.8) | - | - | 46 (58.2) | 33 (41.8) | - | - |
| | | 5-7 | 64 (60.4) | 42 (39.6) | 0.67 (0.37-1.21, p=0.187) | 0.51 (0.23-1.12, p=0.095) | 53 (50.0) | 53 (50.0) | 0.65 (0.36-1.16, p=0.147) | 0.80 (0.31-2.00, p=0.629) | 64 (60.4) | 42 (39.6) | 0.91 (0.51-1.66, p=0.768) | 0.80 (0.37-1.70, p=0.555) |
| | | 8-10 | 18 (54.5) | 15 (45.5) | 0.85 (0.37-1.93, p=0.706) | 0.77 (0.27-2.21, p=0.627) | 16 (48.5) | 17 (51.5) | 0.69 (0.30-1.56, p=0.367) | 0.92 (0.24-3.58, p=0.900) | 21 (65.6) | 11 (34.4) | 0.73 (0.30-1.70, p=0.471) | 0.55 (0.18-1.65, p=0.296) |
| | | 11-20 | 5 (62.5) | 3 (37.5) | 0.62 (0.12-2.68, p=0.525) | 0.55 (0.07-3.89, p=0.545) | 3 (37.5) | 5 (62.5) | 1.08 (0.25-5.55, p=0.923) | 2.30 (0.30-22.78, p=0.434) | 6 (75.0) | 2 (25.0) | 0.46 (0.07-2.16, p=0.366) | 0.29 (0.03-1.91, p=0.223) |
| | Had TB previously | No | 116 (57.4) | 86 (42.6) | - | - | 93 (46.0) | 109 (54.0) | - | - | 124 (61.7) | 77 (38.3) | - | - |
| | | Yes | 11 (45.8) | 13 (54.2) | 1.59 (0.68-3.80, p=0.282) | 0.88 (0.29-2.68, p=0.821) | 10 (41.7) | 14 (58.3) | 1.19 (0.51-2.89, p=0.685) | 1.08 (0.27-4.67, p=0.915) | 13 (54.2) | 11 (45.8) | 1.36 (0.57-3.20, p=0.476) | 1.55 (0.50-4.78, p=0.442) |
| | Family History of TB | No | 104 (55.3) | 84 (44.7) | - | - | 88 (46.8) | 100 (53.2) | - | - | 119 (63.6) | 68 (36.4) | - | - |
| | | Yes | 23 (60.5) | 15 (39.5) | 0.81 (0.39-1.63, p=0.556) | 0.74 (0.28-1.95, p=0.545) | 15 (39.5) | 23 (60.5) | 1.35 (0.67-2.79, p=0.409) | 1.41 (0.48-4.37, p=0.537) | 18 (47.4) | 20 (52.6) | 1.94 (0.96-3.96, p=0.064) | 2.58 (1.00-6.81, p=0.052) |
| | Heard of TB | No | 34 (61.8) | 21 (38.2) | - | - | 22 (40.0) | 33 (60.0) | - | - | 34 (61.8) | 21 (38.2) | - | - |
| | | Yes | 93 (54.4) | 78 (45.6) | 1.36 (0.73-2.56, p=0.335) | 1.70 (0.69-4.28, p=0.252) | 81 (47.4) | 90 (52.6) | 0.74 (0.40-1.37, p=0.341) | 0.96 (0.34-2.62, p=0.930) | 103 (60.6) | 67 (39.4) | 1.05 (0.57-1.99, p=0.871) | 0.48 (0.19-1.20, p=0.121) |
| | Self-medication for TB | No | 102 (56.0) | 80 (44.0) | - | - | 88 (48.4) | 94 (51.6) | - | - | 109 (60.2) | 72 (39.8) | - | - |
| | | Yes | 25 (56.8) | 19 (43.2) | 0.97 (0.49-1.88, p=0.926) | 1.14 (0.44-2.96, p=0.788) | 15 (34.1) | 29 (65.9) | 1.81 (0.92-3.68, p=0.091) | 2.23 (0.76-7.16, p=0.158) | 28 (63.6) | 16 (36.4) | 0.87 (0.43-1.70, p=0.677) | 1.06 (0.38-2.88, p=0.903) |
| Community (societal level) | Stigma associated with TB | Yes/Uncertain | 65 (56.0) | 51 (44.0) | - | - | 61 (52.6) | 55 (47.4) | - | - | 70 (60.3) | 46 (39.7) | - | - |
| | | No | 62 (56.4) | 48 (43.6) | 0.99 (0.58-1.67, p=0.960) | 0.88 (0.40-1.92, p=0.751) | 42 (38.2) | 68 (61.8) | 1.80 (1.06-3.06, p=0.030) | 1.75 (0.73-4.27, p=0.212) | 67 (61.5) | 42 (38.5) | 0.95 (0.56-1.63, p=0.863) | 1.18 (0.55-2.59, p=0.671) |

| | | | | | | | | | | | | | | |
|---|---|---|---|---|---|---|---|---|---|---|---|---|---|---|
| Structural barriers | HFs visited for this illness | Dispensary | 77 (63.1) | 45 (36.9) | - | - | 37 (30.3) | 85 (69.7) | - | - | 73 (60.3) | 48 (39.7) | - | - |
| | | District Hospital | 16 (69.6) | 7 (30.4) | 0.75 (0.27-1.90, p=0.555) | 1.01 (0.27-3.61, p=0.992) | 7 (30.4) | 16 (69.6) | 0.99 (0.39-2.77, p=0.992) | 1.82 (0.54-6.61, p=0.348) | 12 (52.2) | 11 (47.8) | 1.39 (0.56-3.43, p=0.467) | 0.99 (0.28-3.37, p=0.990) |
| | | Health Center/Others | 3 (50.0) | 3 (50.0) | 1.71 (0.31-9.59, p=0.521) | 1.40 (0.15-12.72, p=0.758) | 4 (66.7) | 2 (33.3) | 0.22 (0.03-1.17, p=0.086) | 0.73 (0.05-9.98, p=0.811) | 4 (66.7) | 2 (33.3) | 0.76 (0.10-4.05, p=0.757) | 0.36 (0.03-3.13, p=0.363) |
| | | Private Hospital | 2 (66.7) | 1 (33.3) | 0.86 (0.04-9.17, p=0.900) | 0.27 (0.01-4.74, p=0.386) | 2 (66.7) | 1 (33.3) | 0.22 (0.01-2.34, p=0.219) | 0.11 (0.00-2.17, p=0.160) | 3 (100.0) | 0 (0.0) | - | - |
| | | Regional Hospital | 29 (40.3) | 43 (59.7) | 2.54 (1.40-4.65, p=0.002) | 1.63 (0.63-4.24, p=0.309) | 53 (73.6) | 19 (26.4) | 0.16 (0.08-0.29, p<0.001) | 0.48 (0.17-1.35, p=0.161) | 45 (62.5) | 27 (37.5) | 0.91 (0.50-1.66, p=0.765) | 0.78 (0.28-2.10, p=0.628) |
| | Number of HFs visited | One | 46 (57.5) | 34 (42.5) | - | - | 32 (40.0) | 48 (60.0) | - | - | 47 (59.5) | 32 (40.5) | - | - |
| | | Two | 54 (66.7) | 27 (33.3) | 0.68 (0.35-1.28, p=0.231) | 0.78 (0.22-2.74, p=0.693) | 24 (29.6) | 57 (70.4) | 1.58 (0.83-3.07, p=0.168) | 1.66 (0.47-6.06, p=0.438) | 51 (63.0) | 30 (37.0) | 0.86 (0.46-1.63, p=0.653) | 0.83 (0.23-2.89, p=0.772) |
| | | Three | 8 (57.1) | 6 (42.9) | 1.01 (0.31-3.19, p=0.980) | 0.70 (0.08-5.82, p=0.743) | 2 (14.3) | 12 (85.7) | 4.00 (1.00-26.81, p=0.082) | 2.39 (0.23-30.76, p=0.477) | 7 (50.0) | 7 (50.0) | 1.47 (0.46-4.68, p=0.509) | 2.27 (0.27-20.71, p=0.456) |
| | | Four | 3 (75.0) | 1 (25.0) | 0.45 (0.02-3.70, p=0.499) | 1.24 (0.03-36.64, p=0.900) | 1 (25.0) | 3 (75.0) | 2.00 (0.24-41.41, p=0.556) | 4.34 (0.17-191.56, p=0.396) | 3 (75.0) | 1 (25.0) | 0.49 (0.02-4.02, p=0.544) | 0.29 (0.01-8.83, p=0.491) |
| | | Missing | 16 (34.0) | 31 (66.0) | 2.62 (1.25-5.64, p=0.012) | 3.26 (0.51-24.22, p=0.222) | 44 (93.6) | 3 (6.4) | 0.05 (0.01-0.14, p<0.001) | - | 29 (61.7) | 18 (38.3) | 0.91 (0.43-1.90, p=0.806) | 0.41 (0.04-2.69, p=0.379) |
| | Times of visits | One | 33 (52.4) | 30 (47.6) | - | - | 31 (49.2) | 32 (50.8) | - | - | 41 (65.1) | 22 (34.9) | - | - |
| | | Two | 57 (64.8) | 31 (35.2) | 0.60 (0.31-1.16, p=0.127) | 0.73 (0.21-2.53, p=0.613) | 28 (31.8) | 60 (68.2) | 2.08 (1.07-4.07, p=0.032) | 0.57 (0.16-1.94, p=0.371) | 52 (59.8) | 35 (40.2) | 1.25 (0.64-2.48, p=0.509) | 0.73 (0.20-2.61, p=0.629) |
| | | Three | 13 (59.1) | 9 (40.9) | 0.76 (0.28-2.02, p=0.587) | 1.31 (0.19-8.71, p=0.783) | 3 (13.6) | 19 (86.4) | 6.14 (1.86-27.99, p=0.007) | 2.24 (0.27-23.99, p=0.474) | 14 (63.6) | 8 (36.4) | 1.06 (0.37-2.89, p=0.903) | 0.28 (0.04-1.97, p=0.207) |
| | | Four | 11 (73.3) | 4 (26.7) | 0.40 (0.10-1.31, p=0.150) | 0.63 (0.05-6.40, p=0.706) | 6 (40.0) | 9 (60.0) | 1.45 (0.47-4.79, p=0.522) | 0.38 (0.03-3.77, p=0.413) | 8 (53.3) | 7 (46.7) | 1.63 (0.51-5.14, p=0.400) | 1.43 (0.11-16.56, p=0.778) |
| | | Missing | 13 (34.2) | 25 (65.8) | 2.12 (0.93-4.97, p=0.078) | 0.70 (0.09-4.52, p=0.713) | 35 (92.1) | 3 (7.9) | 0.08 (0.02-0.26, p<0.001) | - | 22 (57.9) | 16 (42.1) | 1.36 (0.59-3.10, p=0.471) | 2.29 (0.38-19.56, p=0.393) |
| | Travel time to the nearest HF | Below 30 min | 44 (54.3) | 37 (45.7) | - | - | 38 (46.9) | 43 (53.1) | - | - | 48 (59.3) | 33 (40.7) | - | - |
| | | Between 30- 60 min | 55 (56.7) | 42 (43.3) | 0.91 (0.50-1.65, p=0.750) | 0.97 (0.40-2.34, p=0.952) | 45 (46.4) | 52 (53.6) | 1.02 (0.56-1.85, p=0.945) | 0.93 (0.34-2.48, p=0.877) | 63 (65.6) | 33 (34.4) | 0.76 (0.41-1.40, p=0.383) | 0.69 (0.29-1.64, p=0.405) |
| | | Above 60 min | 28 (58.3) | 20 (41.7) | 0.85 (0.41-1.74, p=0.657) | 1.16 (0.39-3.57, p=0.791) | 20 (41.7) | 28 (58.3) | 1.24 (0.60-2.56, p=0.563) | 1.04 (0.28-3.91, p=0.951) | 26 (54.2) | 22 (45.8) | 1.23 (0.60-2.53, p=0.572) | 0.99 (0.32-3.04, p=0.983) |
| | Travel time to this HF | Below 30 min | 64 (55.7) | 51 (44.3) | - | - | 61 (53.0) | 54 (47.0) | - | - | 74 (64.3) | 41 (35.7) | - | - |
| | | Between 30- 60 min | 26 (48.1) | 28 (51.9) | 1.35 (0.71-2.59, p=0.363) | 1.48 (0.57-3.86, p=0.419) | 23 (42.6) | 31 (57.4) | 1.52 (0.80-2.94, p=0.206) | 0.74 (0.25-2.19, p=0.589) | 32 (59.3) | 22 (40.7) | 1.24 (0.64-2.41, p=0.524) | 2.06 (0.79-5.48, p=0.141) |
| | | Above 60 min | 37 (64.9) | 20 (35.1) | 0.68 (0.35-1.30, p=0.247) | 0.76 (0.27-2.16, p=0.608) | 19 (33.3) | 38 (66.7) | 2.26 (1.18-4.44, p=0.016) | 1.58 (0.50-5.15, p=0.439) | 31 (55.4) | 25 (44.6) | 1.46 (0.76-2.79, p=0.258) | 2.62 (0.92-7.72, p=0.074) |
| | Travel & Wait time to this HF | Below 10 min | 48 (46.6) | 55 (53.4) | - | - | 50 (48.5) | 53 (51.5) | - | - | 61 (59.2) | 42 (40.8) | - | - |
| | | 10- 60 min | 22 (57.9) | 16 (42.1) | 0.63 (0.30-1.34, p=0.236) | 0.75 (0.25-2.18, p=0.591) | 12 (31.6) | 26 (68.4) | 2.04 (0.95-4.61, p=0.074) | 0.81 (0.22-3.05, p=0.751) | 27 (71.1) | 11 (28.9) | 0.59 (0.26-1.30, p=0.201) | 0.18 (0.05-0.58, p=0.005) |
| | | 60- 120 min | 38 (70.4) | 16 (29.6) | 0.37 (0.18-0.73, p=0.005) | 0.89 (0.24-3.24, p=0.861) | 29 (53.7) | 25 (46.3) | 0.81 (0.42-1.57, p=0.539) | 0.22 (0.05-1.01, p=0.053) | 27 (50.0) | 27 (50.0) | 1.45 (0.75-2.83, p=0.270) | 0.22 (0.05-0.83, p=0.031) |
| | | Above 120 min | 19 (61.3) | 12 (38.7) | 0.55 (0.24-1.24, p=0.154) | 1.03 (0.23-4.52, p=0.973) | 12 (38.7) | 19 (61.3) | 1.49 (0.66-3.46, p=0.337) | 0.35 (0.06-1.82, p=0.212) | 22 (73.3) | 8 (26.7) | 0.53 (0.20-1.26, p=0.164) | 0.04 (0.01-0.23, p=0.001) |

**S Table 8: Factors associated with prolonged time to diagnosis among children presumptive EPTB patients.**

| | | | By Patient Delay | | | | By Health system level Delay Phase I | | | | By Health System Level Delay Phase II | | | |
|---|---|---|---|---|---|---|---|---|---|---|---|---|---|---|
| | Subgroups | Categories | Below Median PD | Above Median PD | OR (univariable) | OR (multivariable) | Below Median SD | Above Median SD | OR (univariable) | OR (multivariable) | Below Median SD | Above Median SD | OR (univariable) | OR (multivariable) |
| Individual (patient-level) | By EPTB case confirmation status | EPTB negative | 28 (77.8) | 8 (22.2) | - | - | 21 (58.3) | 15 (41.7) | - | - | 20 (55.6) | 16 (44.4) | - | - |
| | | EPTB patient | 11 (47.8) | 12 (52.2) | 3.82 (1.25-12.35, p=0.021) | 27.91 (4.15-315.40, p=0.002) | 12 (52.2) | 11 (47.8) | 1.28 (0.45-3.71, p=0.642) | 0.91 (0.16-4.63, p=0.907) | 13 (56.5) | 10 (43.5) | 0.96 (0.33-2.76, p=0.942) | 1.33 (0.35-5.21, p=0.673) |
| | Gender | Female | 20 (69.0) | 9 (31.0) | - | - | 14 (48.3) | 15 (51.7) | - | - | 18 (62.1) | 11 (37.9) | - | - |
| | | Male | 19 (63.3) | 11 (36.7) | 1.29 (0.44-3.87, p=0.648) | 2.91 (0.54-19.96, p=0.236) | 19 (63.3) | 11 (36.7) | 0.54 (0.19-1.52, p=0.246) | 0.90 (0.17-4.66, p=0.902) | 15 (50.0) | 15 (50.0) | 1.64 (0.58-4.70, p=0.352) | 1.72 (0.47-6.66, p=0.417) |
| | Education levels | Primary or below | 33 (63.5) | 19 (36.5) | - | - | 31 (59.6) | 21 (40.4) | - | - | 28 (53.8) | 24 (46.2) | - | - |
| | | Middle or Secondary | 6 (85.7) | 1 (14.3) | 0.29 (0.01-1.87, p=0.267) | 0.02 (0.00-0.55, p=0.037) | 2 (28.6) | 5 (71.4) | 3.69 (0.72-27.49, p=0.139) | 15.75 (1.43-261.28, p=0.034) | 5 (71.4) | 2 (28.6) | 0.47 (0.06-2.38, p=0.387) | 0.71 (0.08-5.21, p=0.744) |
| | Salary Categories | Low income | 4 (66.7) | 2 (33.3) | - | - | 4 (66.7) | 2 (33.3) | - | - | 4 (66.7) | 2 (33.3) | - | - |
| | | Middle income | 18 (81.8) | 4 (18.2) | 0.44 (0.06-4.00, p=0.430) | 0.69 (0.04-11.64, p=0.788) | 15 (68.2) | 7 (31.8) | 0.93 (0.14-7.90, p=0.944) | 0.52 (0.04-9.11, p=0.635) | 11 (50.0) | 11 (50.0) | 2.00 (0.32-16.67, p=0.473) | 3.47 (0.45-37.17, p=0.254) |
| | | High income | 17 (54.8) | 14 (45.2) | 1.65 (0.28-13.17, p=0.595) | 6.37 (0.49-130.85, p=0.185) | 14 (45.2) | 17 (54.8) | 2.43 (0.41-19.44, p=0.344) | 3.32 (0.29-59.86, p=0.368) | 18 (58.1) | 13 (41.9) | 1.44 (0.24-11.56, p=0.695) | 2.42 (0.32-24.61, p=0.407) |
| | Number of family members | 1-4 | 6 (42.9) | 8 (57.1) | - | - | 3 (21.4) | 11 (78.6) | - | - | 7 (50.0) | 7 (50.0) | - | - |
| | | 5-7 | 26 (74.3) | 9 (25.7) | 0.26 (0.07-0.94, p=0.042) | 0.24 (0.04-1.30, p=0.105) | 24 (68.6) | 11 (31.4) | 0.13 (0.02-0.49, p=0.005) | 0.04 (0.00-0.28, p=0.004) | 22 (62.9) | 13 (37.1) | 0.59 (0.17-2.09, p=0.410) | 0.63 (0.15-2.59, p=0.521) |
| | | 8-10 | 6 (75.0) | 2 (25.0) | 0.25 (0.03-1.54, p=0.157) | 0.08 (0.00-0.76, p=0.042) | 5 (62.5) | 3 (37.5) | 0.16 (0.02-1.03, p=0.064) | 0.16 (0.01-1.51, p=0.126) | 3 (37.5) | 5 (62.5) | 1.67 (0.29-10.86, p=0.572) | 1.55 (0.24-11.21, p=0.646) |
| | | 11-20 | 1 (50.0) | 1 (50.0) | 0.75 (0.03-21.64, p=0.849) | 2.22 (0.03-263.60, p=0.713) | 1 (50.0) | 1 (50.0) | 0.27 (0.01-8.27, p=0.404) | 0.06 (0.00-3.40, p=0.166) | 1 (50.0) | 1 (50.0) | 1.00 (0.03-28.82, p=1.000) | 3.04 (0.08-139.71, p=0.525) |
| | Heard of TB | No | 38 (67.9) | 18 (32.1) | - | - | 31 (55.4) | 25 (44.6) | - | - | 31 (55.4) | 25 (44.6) | - | - |
| | | Yes | 1 (33.3) | 2 (66.7) | 4.22 (0.38-94.43, p=0.252) | 1.14 (0.03-79.40, p=0.945) | 2 (66.7) | 1 (33.3) | 0.62 (0.03-6.84, p=0.703) | 2.34 (0.06-61.72, p=0.606) | 2 (66.7) | 1 (33.3) | 0.62 (0.03-6.84, p=0.703) | 0.43 (0.02-6.68, p=0.553) |
| | Had TB previously | No | 35 (67.3) | 17 (32.7) | - | - | 29 (55.8) | 23 (44.2) | - | - | 30 (57.7) | 22 (42.3) | - | - |
| | | Yes | 4 (57.1) | 3 (42.9) | 1.54 (0.28-7.78, p=0.596) | 0.91 (0.07-11.43, p=0.938) | 4 (57.1) | 3 (42.9) | 0.95 (0.17-4.71, p=0.945) | 0.78 (0.06-7.01, p=0.833) | 3 (42.9) | 4 (57.1) | 1.82 (0.37-10.02, p=0.463) | 2.22 (0.35-16.27, p=0.402) |
| | Family History of TB | No | 13 (59.1) | 9 (40.9) | - | - | 13 (59.1) | 9 (40.9) | - | - | 12 (54.5) | 10 (45.5) | - | - |
| | | Yes | 26 (70.3) | 11 (29.7) | 0.61 (0.20-1.86, p=0.382) | 0.36 (0.06-1.95, p=0.248) | 20 (54.1) | 17 (45.9) | 1.23 (0.42-3.65, p=0.706) | 7.91 (1.46-63.53, p=0.028) | 21 (56.8) | 16 (43.2) | 0.91 (0.31-2.67, p=0.869) | 0.90 (0.25-3.19, p=0.868) |
| Community (societal level) | Stigma associated with TB | Yes/Uncertain | 23 (69.7) | 10 (30.3) | - | - | 23 (69.7) | 10 (30.3) | - | - | 18 (54.5) | 15 (45.5) | - | - |
| | | No | 16 (61.5) | 10 (38.5) | 1.44 (0.48-4.31, p=0.512) | 1.82 (0.34-10.53, p=0.485) | 10 (38.5) | 16 (61.5) | 3.68 (1.27-11.29, p=0.019) | 7.85 (1.69-48.57, p=0.014) | 15 (57.7) | 11 (42.3) | 0.88 (0.31-2.48, p=0.809) | 0.75 (0.21-2.55, p=0.641) |

*p-value corresponds to the use of the Pearson chi-square test.
