# Supplementary material for: Factors associated with the delay in diagnosis of extrapulmonary tuberculosis at the patient and health system level: A study from a rural setting in India
Source: PLoS One. 2025 Jan 7;20(1):e0316273. doi: 10.1371/journal.pone.0316273 (PMC11706485; doi:10.1371/journal.pone.0316273)
Supplement: S2 Text — (PDF) [file pone.0316273.s002.pdf]

Study Questionnaire

STUDY: IMPROVED DIAGNOSIS OF EXTRAPULMONARY TB

PATIENT REGISTRATION FORM

QUESTIONNAIRE–15 years and above

Date:

Paramedic/ Doctors (who is interviewing the patient)**:**

Hospital: Ruxamaniben Deepchand Gardi Medical College, Ujjain (MP)

Department: □ 1)OPD □ 2)IPD 3)Department 4)Registration No.

Extrapulmonary TB Suspect: □ Yes □ No

INFORMED CONSENT

Informed consent (Part 1)

□ **Yes** □ **No**

If Yes, complete sections A-F below.

SECTION- A

PATIENT IDENTIFICATION

Name of patient: ______________________________ **Study Number(three digit e.g.000)**: _______

Age (years):________

Gender: □ 1) Male □2) Female

Respondent: □ 1) Patient □2) Parent □3) Spouse 4) Child □5) Other, relative/friend

Address: District __________City____________Village/Street/House___________________________

Contact No.________________

SECTION- B

PERSONAL INFORMATION

1. **Marital status:**

□1) Single □2) Married □3) Widow/widower □4) Separated

□ 5) Divorced □6) Other, please specify____________

2. **Level of education:**

□1) No formal education □2) Not completed primary school

□3) Completed primary school 4) Completed middle school

□5) Completed secondary school □ 6) Above secondary school

□7) Adult education □ 8) Others (Please mention)______

3. **Religion**:

□1)Hindu 2)Muslim □ 3)Christian □ 4)Other, please mention____________

4. **Do you use chewable tobacco** (e.g.Pan, Niswar, Gutka)**:**□ Yes □ No ____weeks/months /years
5. **Do you smoke cigarettes/ Huka (water pipe), alcohol:**□ Yes □ No ____weeks/months /years

SECTION- C

PAST MEDICAL HISTORY

6. **Do you have any of these diseases?**

COPD: □ Yes □ No

Renal Disease: □ Yes □ No

Liver Diseases: □ Yes □ No

Diabetes Mellitus: □ Yes □ No

Hypertension: □ Yes □ No

Other: □ Yes □ No

Describe other: ______________________________

____

SECTION-D

HEALTH SEEKING BEHAVIOUR & DIAGNOSTIC DELAY

Health seeking behavior of TB patients

Please remind the patient that this survey is confidential.

7. Please ask if the patient has experienced any of the following symptoms

7.1. General Symptoms

Fever: □ Yes □ No ____weeks/months

What kind of fever do you have? □1) High-grade □2) Low-grade

When do you have fever? □1)Morning □2)Day-time □3)Evening □4)Night □5)all day

Loss of weight: □ Yes □ No ____weeks/months
Loss of appetite: □ Yes □ No ____weeks/months
Night Sweat: □ Yes □ No ____weeks/months
Fatigue: □ Yes □ No ____weeks/months
Amenorrhea(female only): □ Yes □ No ____weeks/months
Body weakness: □ Yes □ No ____weeks/months
Frequent cold: □ Yes □ No ____weeks/months
Neck mass: □ Yes □ No ____weeks/months
Other: □ Yes □ No ____weeks/months
Describe:________________________

7.2. Respiratory Symptoms

Cough: □ Yes □ No ___weeks/months
Sputum: □ Yes □ No ___weeks/months
Cough with Sputum: □ Yes □ No ___weeks/months
Blood with Sputum: □ Yes □ No ___weeks/months
Chest pain: □ Yes □ No ___weeks/months
Difficulty in breathing: □ Yes □ No ___weeks/months

7.3. Abdominal Symptoms
Swelling of/in stomach: □ Yes □ No ___weeks/months
Fullness of stomach: □ Yes □ No ___weeks/months
Vomiting: □ Yes □ No ___weeks/months
Diarrhea: □ Yes □ No ___weeks/months
Other: □ Yes □ No ___weeks/months
Describe other: ____

7.4. Neurological Symptoms
Headache: □ Yes □ No ___weeks/months
Photophobia: □ Yes □ No ___weeks/months
Vomiting: □ Yes □ No ___weeks/months
Dizziness: □ Yes □ No ___weeks/months
Vertigo: □ Yes □ No ___weeks/months
Weakness/Numbness of extremity: □ Yes □ No ___weeks/months
Visual disturbance: □ Yes □ No ___weeks/months

Other: □ Yes □ No ___weeks/months
Describe other: ______________________________

8. What were the major symptoms that first made you seek care?

□1)Prolong Cough □2)Blood with sputum □3)Breathlessness
□4)Chest pain □5)Fever □ 6)Weight loss
□7)Fatigue\Weakness 8)Loss of appetite □ 9) Night sweats
□10)Bone pain 11)Lymph node swelling □ 12)Diarrhoea
□13)Abdominal pain □14)others (specify) _________________

9. When did you first notice the symptoms?

____

10. Did you practice any self-medication before you sought care? □ Yes □ No

11. How long did you experience these symptoms before you went to seek treatment?

______________________ (days/ weeks)

12. How many different places did you go to seek help for the current symptoms? Number?______________and type of places?_________________

13. How many times have you visited health facilities with the same symptoms before?

□ 1)First visit □ 2)Second visit □ 3)Third visit

□ 4) > 3 visits 5)don't remember

14. Which place did you first seek care for your symptoms?

□1)Tertiary Care Hospital □2)District hospital □ 3)Rural health center

□4)Private Hospital/clinic □5)Traditional healer 6)Pharmacy

□7)other, please specify____________________________________

15. What kind of diagnosis did you receive for your illness? ________________________

16. Were any tests done at the first medical service?

□ Yes □ No

17. What type of tests?

□1)Blood test □2)Urine test □3)Sputum □4)X-ray

□5)Others, please specify________________________________

18. Did you take the results back to the doctor?

□ Yes □ No

19. Could you estimate the total cost for the previous visits/investigations related to your current illness?

Admission __________________________INR
Consultations __________________________INR
Medication __________________________INR
Laboratory tests/X-ray/CT_________________________INR

Transportation ___________________________INR

20.Who has referred you to Ruxamaniben Deepchand Gardi Medical College, Ujjain (MP)

□1)Self 2)Traditional healers □3)Religious leaders
□4)Pharmacy/drug shop 5)Village health worker □6)Government dispensary
□7)Government health center 8)Government hospital □9)Private dispensary/hospital
□10)Charitable/NGO 11)Member of the family □12)Other____________________

20. Before today, had you heard of the disease tuberculosis?

□ Yes □ No

21. Do you have any one in your family who has been diagnosed with TB before?

Yes □ No

If yes? From where he/she has taken treatment? ______________________________

22. Do you drink un boiled milk?

□ Yes □ No

23. Do people in your community stigmatize/ discriminate person having tuberculosis?

□1)Yes □2)No □3)Uncertain

If yes, why? ___________________________________

24. Is there anything that would make it easier for people with tuberculosis to get treatment, not just in this clinic, but in other health facilities?

□ 1)Yes □2)No □3)Uncertain

If yes, what could be done? ____

24. (1)What fears do others have about TB that prevents them from seeking medical advice?

____

SECTION- E

EXAMINATION

25. Physical signs

25.1. <u>General</u>

Weight: ______K.g.
Temperature: ______ Deg. Centigrade
Pulse rate: ______b.p.m
Blood pressure: ___________
Pallor: □ Yes □ No
Finger clubbing: □ Yes □ No
BCG scar: □ Yes □ No
Other: □ Yes □ No

25.2. <u>Lymph nodes</u>

Lymph node enlargement: □ Yes □ No

Matted: ☐ Yes ☐ No
Painful: ☐ Yes ☐ No
Discharge/Sinus: ☐ Yes ☐ No

Please draw enlarged lymph nodes or other findings:

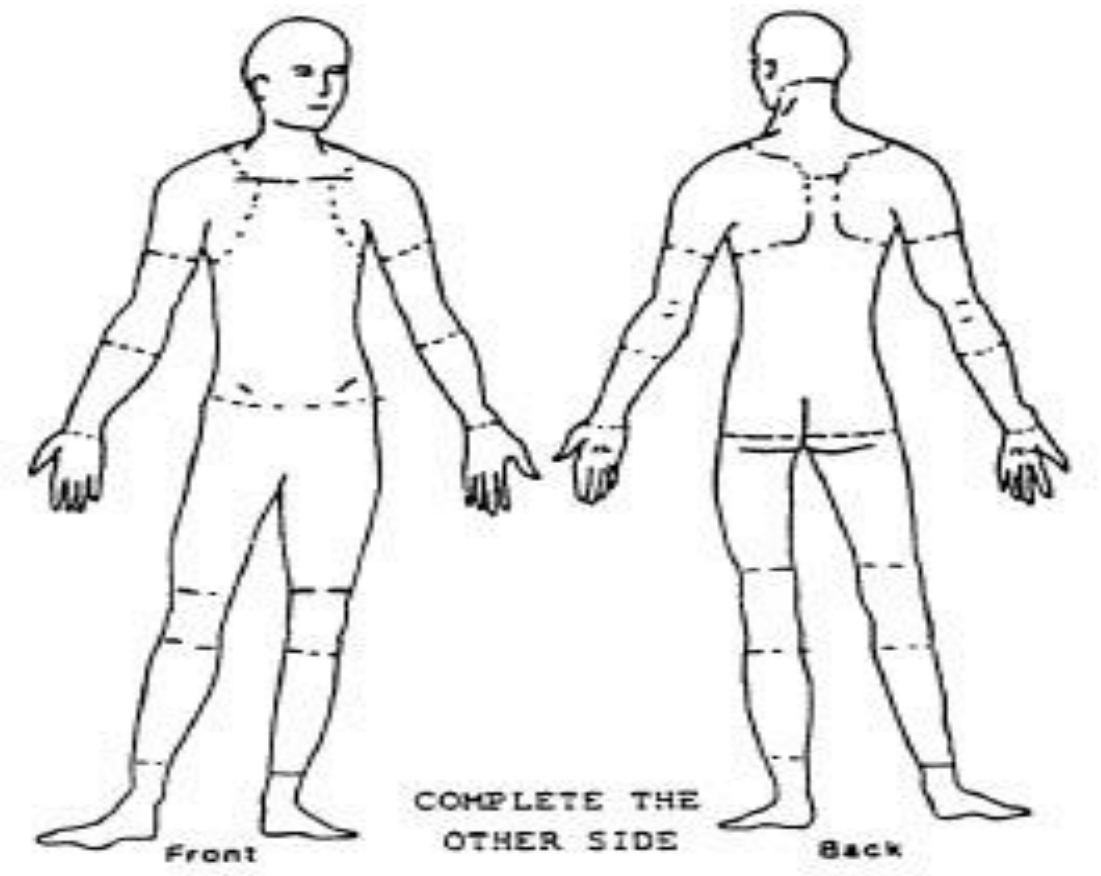

25.3. <u>Other Clinical Findings (as documented by the physicians/specialist)</u>__________________

____

SECTION- F

INVESTIGATIONS

26. Blood sample
Hb _______________ ESR ______________
White blood cell count: ____
LFT____
HIV/ HBsAg____

27. Bacteriology result
27.1. <u>Sputum examination</u>

AFB microscopy
Date (day.month.year) **Appearance *** **Neg.** + ++ +++

Sample 1 (Spot-1)____

Sample 2 (Morning)____

*visual appearance (blood stained, muo-purulent, saliva)

MTB Culture ☐1) Positive 0) Negative

Date of positive culture (day, month, year):__________________________

GeneXpert ☐1) Positive ☐0) Negative

RIF resistant ☐Yes ☐ No

27.2. **<u>Other samples investigated</u>** (pleura fluid, ascites, lymph node biopsies, FNA, CSF)

Material: _______________________________________

Laboratory serial number: ___________________

AFB microscopy: ☐1)Positive ☐0)Negative

Cytology/histology: ____

____

MTB Culture: ☐ 1)Positive ☐0)Negative

ADA ……………….(IU/L)

MTP64: ☐ 1)Positive ☐0)Negative

Biochemical tests: 1)Protein __________ 2)Glucose__________3)Cell count: __________________

GeneXpert: ☐ 1)Positive ☐0)Negative

RIF res ☐ No ☐ Yes

Gram stain:__________________________________ **Bact. Culture:**______________________________

Other tests: ____

28. Other Investigations

28.1. X-Ray Chest

____

____

28.2. Sonography/CT scan

____

____

28.3. Any other (specify): ____

SECTION-G

PATIENT WHO ARE REGISTERED FOR EPTB TREATMENT & FOLLOW-UP

TB Registration Number: _________________

Final Diagnosis: _________________________________

Patient Condition at the time of follow-up (Clinically improved, Not improved, Somewhat improved):

Follow-up 1.	Follow-up 3.	Follow-up 5.
Follow-up 2.	Follow-up 4.	Follow-up 6.

SECTION- H

QUALITY OF LIFE

29. Quality of Life (at time of registration):

29.1. Are you able to walk?

□1)I have no problem in walking about □2)I have some problem in walking about □3)I am confined to bed

29.2. Are you able to perform usual activity? (such as work, studies, domestic work, etc)

□ 1)I have no problem with performing my usual activity □2)I have some problem with performing my usual activity □ 3)I am unable to perform my usual activity

29.3. Are you having any pain/ discomfort?

□ 1)I have no pain/discomfort □2)I have moderate pain/discomfort □3)I have extreme pain/discomfort

29.4. Are you anxious/ depressed?

□ 1)I am not anxious/ depressed □2)I am moderately anxious/ depressed □3)I am extremely anxious/ depressed

SECTION-I<br>PATIENT AND HOUSEHOLD COSTS

Estimate of the patient income level

30. How long does it take you to go to the nearest health facility?

□ 1)Less than 30 minutes□2) between 30 minutes and one hour□ 3)More than one hour

31. How far is this hospital to your home (in Kilometers) ________________

32. How long (on average) does it take you to this health facility, waiting for your consultation and finally returning to your home\workplace? ________________ Hours

33. How did you get to this health facility?

□ 1)Walked □ 2)Bicycle □3)Motorcycle □ 4)Private car □5)Rikshaw/taxi □ 6)Bus

34. If you have to take a public transport (e.g. Rikshaw /taxi/ bus)how much (on average) does it cost you to come to the clinic? ________________INR.

35. Do you have to make some special arrangements at home before coming to the Hospital? For example: To look after your children back home in your absence, any disabled persons, pregnant women or any job related arrangements?

□ Yes □ No □ Uncertain

If yes, what arrangements?__________________________

36. What is your main occupation (past twelve months)?

□ 1)Employed by government □ 2)Employed private

□ 3)Self-employed (mention the self-employment) 4)Student 5)Housewife □ 6)Other_________________

37. What is the main source of income of you and your house holds?

□ 1)Employment (Govt or private) □2)Pensions

□ 3)Crop production □ 4)Livestock □5)Fishing

□ 6)Hunting / bee-keeping 7)Poultry □8)Farm wage

□ 9)Other agricultural activity □ 10)Wages (government) □11)Wages (private)

□ 12)Monetary savings (interest) 13) Property (rentals) □14)Self-employed payments (merchant) □15)Other Specify____________________________

38. How much did (NAME) earn (money) for the activities stated on average in the past 12 months? This should include not only salary or cash income: but also the value of goods produced or traded for other goods and services.

____

BetweenINR:

□ 1)Less than 10,000

□ 2)10,000 – 20,000

□ 3)21,000-30,000

□ 4)31,000 – 40,000

□ 5)41,000-50,000

□ 6)More than 50,000

39. Do you have reduced working capacity due to your current illness?
□ 1)Yes, completely stopped working □2)Yes, working but with reduced capacity
□ 3)Working as normal

40.1. If yes, what is the level of this reduction in working capacity (percentage of total working capacity before illness)? _________%

40.2. How many days have you faced this reduced working capacity)?______________ days

40.3. Have any member of your household stopped working or reduced their work capacity because of your illness?

· Yes · No

If yes, how much reduced working capacity? _____________________%

If yes, for how long? _______________days
41. Have you/or any member of your household lost any wages or income because of your illness?
□ Yes □ No □ Uncertain
If yes, how much____________________

42. Do you own a house?
□ 1)Yes □2)Renting a house □3) Living with relatives /friends □ 4)Homeless

43. How many people live in your household: _________________ (number of people)

44. What is the main source of drinking water for members of your household?
□ 1)Piped water 1=Piped into dwelling 2= Piped into yard/plot 3=Public tap 4=Neighbors' tap
□ 2)Hand Pump
□ 3)Water supplied by Tanker/Truck
□ 4)Water from open well
□ 5)Tube well/Turbine
□ 6)Running water 1=spring; 2=river/stream; 3=pond/Lake; 4=Dam
□ 7)Rain water
□ 8)Water vendor
□ 9)Bottled water
□ 10)Others Specify ________________________

45. What kind of toilet facilities do members of your household usually use?
□ 1)Flush to piped sewer system □2)Flush to septic tank
□ 3)Open Pit □ 4)Ventilated improved pit (VIP) □5)Public Latrine
□ 6)No facility/bush/field □7)other, please specify ______________

46. Does your household have?

 1)Electricity 2)Gas 3)Radio 4)Television
5)Telephone/mobile 6)Iron (either charcoal or electricity) 7)Refrigerator

47. What is the main source of energy for lighting in your household?

 1)Main electricity 2)Solar 3)Gas 4)Kerosene lamp
5)Firewood 6)Candles 7)other, please specify ___________________________

48. What is the main material for the walls of your house or house you are living?

 1)Mud 2)Cement bricks 3)Backed bricks 4)Wood
5)Stones 6)Others Specify___________________________

49. What is the roofing material of your house or house you are living?

 1)Grass/leaves/mud 2)Iron sheets 3)Tiles 4)Concrete/Cement
 5)Others Specify________________

50. Does you or any member of your household own?

 1)A bicycle 2)A motorcycle or motor scooter 3)A car 4)A bank account

51. How many acres of land for farming/grazing are owned by the household?

 Arable land______________acres Land for grazing___________acres

52. How many meals does your household usually have per day?

Meals (in number)?__________________

Informed consent (Part 2- for Blood Dry Spot): 1) Yes 2) No

If Yes, take the blood sample on paper and store as per the guidelines.

SECTION- J

DIABETES SCREENING

53. Pre-diabetic (risk of getting diabetes)

53.1 Do you have a mother or father or brother or sister and/or own child with diabetes?

 Yes No Uncertain

If yes, who _____________________

53.2 BMI of Participant [Use the BMI chart)? ____________ _____

53.3 Has a doctor ever told you that you have high blood pressure, or given you medication for it?

 Yes No Uncertain

53.4 Nationality? ______________________
53.5 Risk calculation (score to establish as pre-diabetic)?__________________

(Use Finnish Scoring chart)

54. Patient is known diabetic (from question 6)?

 Yes No ______ weeks/months/ years

If yes? Are you taking medication for diabetes?

 Yes No If yes? Which medicines?______________

If unknown diabetic?

Screen with Random Blood Glucose (using gluco-meter)

Result of RBG? ___________ (mg/dl)

If RBG ≥ 140-199 mg/dl , perform OGTT (offer 75mg of glucose dissolved in water and check blood sugar after 2 hours)

Result of PPBG (Post prandial blood glucose)? ___________ (mg/dl)

If PPBG <140 mg/dl (normal), if* ≥ 140-199 *mg/dl (pre-diabetic) and if* ≥ 200 mg/dl *refer the patient to physician/ Diabetes Specialist with patient results.

SECTION- K

END OF TREATMENT

55. Quality of Life (at end of treatment duration):

55.1 Are you able to walk?

 1)I have no problem in walking about 2)I have some problem in walking about 3)I am confined to bed

55.2 Are you able to perform usual activity? (such as work, studies, domestic work, etc)

1) have no problem with performing my usual activity 2)I have some problem with performing my usual activity 3)I am unable to perform my usual activity

55.3 Are you having any pain/ discomfort?

 1)I have no pain/discomfort 2)I have moderate pain/discomfort 3)I have extreme pain/discomfort

55.4 Are you anxious/ depressed?

 1)I am not anxious/ depressed 2)I am moderately anxious/ depressed 3)I am extremely anxious/ depressed

56. Response to treatment (at end of treatment duration):

56.1 Presenting complaints (signs and symptoms)?

 1)Settled 2)Somewhat settled 3)Not settled

56.2 Treatment outcome?

 1)Treatment completed 2)Lost to follow-up 3)Treatment Failure 4)Died 5)Not Evaluated `

Under each heading, please tick the ONE box that best describes your health TODAY.

MOBILITY

I have no problems in walking about ❑

I have some problems in walking about ❑

I am confined to bed ❑

SELF-CARE

I have no problems with self-care ❑

I have some problems washing or dressing myself ❑

I am unable to wash or dress myself ❑

USUAL ACTIVITIES (*e.g. work, study, housework, family or leisure activities*)

I have no problems with performing my usual activities ❑

I have some problems with performing my usual activities ❑

I am unable to perform my usual activities ❑

PAIN / DISCOMFORT

I have no pain or discomfort ❑

I have moderate pain or discomfort ❑

I have extreme pain or discomfort ❑

ANXIETY / DEPRESSION

I am not anxious or depressed ❑

I am moderately anxious or depressed ❑

I am extremely anxious or depressed ❑

SAMPLE

- We would like to know how good or bad your health is TODAY.
- This scale is numbered from 0 to 100.
- 100 means the best health you can imagine.
  0 means the worst health you can imagine.
- Mark an X on the scale to indicate how your health is TODAY.
- Now, please write the number you marked on the scale in the box below.

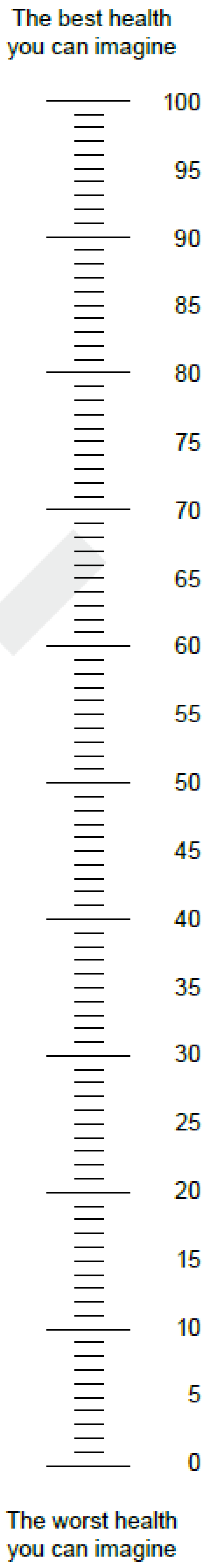

YOUR HEALTH TODAY = [ ]

SAMPLE

In previous versions of the EQ-5D-3L, the numerical scale straddled the EQ VAS (provided in the annex for reference). Users are encouraged to use the latest version of the EQ-5D-3L in new studies.
